# Machine learning of electrophysiological signals for the prediction of ventricular arrhythmias: systematic review and examination of heterogeneity between studies

**DOI:** 10.1016/j.ebiom.2023.104462

**Published:** 2023-02-09

**Authors:** Maarten Z.H. Kolk, Brototo Deb, Samuel Ruipérez-Campillo, Neil K. Bhatia, Paul Clopton, Arthur A.M. Wilde, Sanjiv M. Narayan, Reinoud E. Knops, Fleur V.Y. Tjong

**Affiliations:** a Amsterdam UMC Location University of Amsterdam, Heart Center, Department of Clinical and Experimental Cardiology, Meibergdreef 9, Amsterdam, the Netherlands; b Amsterdam Cardiovascular Sciences, Heart failure & arrhythmias, Amsterdam, The Netherlands; c Department of Medicine and Cardiovascular Institute, Stanford University, Stanford, CA, USA; d Department of Cardiology, Emory University, Atlanta, GA, USA

**Keywords:** Cardiology, Artificial intelligence, Electrocardiography, Systematic review, Meta-analysis, Machine Learning

## Abstract

**Background:**

Ventricular arrhythmia (VA) precipitating sudden cardiac arrest (SCD) is among the most frequent causes of death and pose a high burden on public health systems worldwide. The increasing availability of electrophysiological signals collected through conventional methods (e.g. electrocardiography (ECG)) and digital health technologies (e.g. wearable devices) in combination with novel predictive analytics using machine learning (ML) and deep learning (DL) hold potential for personalised predictions of arrhythmic events.

**Methods:**

This systematic review and exploratory meta-analysis assesses the state-of-the-art of ML/DL models of electrophysiological signals for personalised prediction of malignant VA or SCD, and studies potential causes of bias (PROSPERO, reference: CRD42021283464). Five electronic databases were searched to identify eligible studies. Pooled estimates of the diagnostic odds ratio (DOR) and summary area under the curve (AUROC) were calculated. Meta-analyses were performed separately for studies using publicly available, *ad-hoc* datasets, versus targeted *clinical* data acquisition. Studies were scored on risk of bias by the PROBAST tool.

**Findings:**

2194 studies were identified of which 46 were included in the systematic review and 32 in the meta-analysis. Pooling of individual models demonstrated a summary AUROC of 0.856 (95% CI 0.755–0.909) for short-term (time-to-event up to 72 h) prediction and AUROC of 0.876 (95% CI 0.642–0.980) for long-term prediction (time-to-event up to years). While models developed on *ad-hoc* sets had higher pooled performance (AUROC 0.919, 95% CI 0.867–0.952), they had a high risk of bias related to the re-use and overlap of small *ad-hoc* datasets, choices of ML tool and a lack of external model validation.

**Interpretation:**

ML and DL models appear to accurately predict malignant VA and SCD. However, wide heterogeneity between studies, in part due to small *ad-hoc* datasets and choice of ML model, may reduce the ability to generalise and should be addressed in future studies.

**Funding:**

This publication is part of the project DEEP RISK ICD (with project number 452019308) of the research programme Rubicon which is (partly) financed by the 10.13039/501100003246Dutch Research Council (10.13039/501100003246NWO). This research is partly funded by the 10.13039/100019741Amsterdam Cardiovascular Sciences (personal grant F.V.Y.T).

**Research in context**

**Evidence before this study**

Sudden cardiac deaths (SCD) and malignant ventricular arrhythmias (VA) represent a major public health problem globally. Although risk factors for SCD and malignant VA have been identified (e.g. a left ventricular ejection fraction ≤35%), the majority of events occur in individuals without any risk factors. Currently, there is no effective screening tool to identify *at-risk* individuals of either SCD or malignant VA. The emergence of artificial intelligence (AI) and increasing availability of electrophysiological signals obtained non-invasively using body-surface electrocardiography (ECG), intra-cardiac devices and wearable sensors could facilitate personalised prediction of SCD and malignant VA. We searched the MEDLINE (Ovid), EMBASE (Ovid), Scopus, Web of Science and Cochrane Library Databases electronic databases to identify studies published before August 2021 that developed a machine learning (ML) or deep learning (DL) model for prediction of malignant VA or SCD using electrophysiological signals. We found that the predictive performance of individual ML and DL models were generally high, and in particular ML and DL models derived from publicly available datasets had superior accuracy. However, these studies were characterised by a high risk of bias and methodological limitations that hinder their potential translation to clinical practice.

**Added value of this study**

This systematic review and meta-analysis examines the current state of AI-based models that use electrophysiological signals to predict for SCD and malignant VA. Our systematic assessment of ML and DL models revealed important methodological limitations that could affect the potential uptake of these models. We highlighted aspects necessary for adoption of ML and DL models in clinical practice, including external model validation, targeted model deployment, explainable AI and model transparency.

**Implications of all the available evidence**

Predictive models developed using AI achieve high performance and enable automated and personalised predictions. However, methodological limitations have consequences for the generalisability, clinical utility and reproducibility of these models. In order for research on the intersection of medicine and AI to be relevant and useful in clinical practice, it is essential that future studies adhere to high methodological standards.

## Introduction

Sudden cardiac death (SCD) and out-of-hospital cardiac arrest are often precipitated by ventricular arrhythmias (VA) and account for 400.000 deaths annually in the United States alone.^1^^,^^2^ Risk stratification for SCD and malignant VA in clinical practice is currently based on left ventricular (LV) systolic dysfunction.^3^, ^4^, ^5^ However, LV dysfunction is inadequate as the sole surrogate marker for the underlying dynamic and complex mechanisms responsible for malignant VA.^6^^,^^7^ The majority of patients who suffer an out-of-hospital cardiac arrest or SCD have preserved left ventricular systolic function.^8^^,^^9^ New approaches to predict VA may be enabled by a combination of artificial intelligence (AI) and the increasing availability in electrophysiological signals obtained non-invasively using body-surface electrocardiography (ECG), intra-cardiac devices or wearable sensors. Machine learning (ML) and deep learning (DL) facilitate detection of ECG signatures and patterns that are unrecognizable by the human eye and might indicate sub-clinical pathology.^10^ This extends the traditional identification of specific, often manually extracted features analysed in isolation as predictors of malignant VA and SCD.^11^, ^12^, ^13^, ^14^ Over the past decade, extensive research has been conducted on the use of ML and DL to predict malignant VA and SCD, of which the current state-of-the-art is unclear.^15^, ^16^, ^17^, ^18^ The aim of this systematic review and meta-analysis was to critically evaluate the merits and pooled accuracy of ML and DL models that use electrophysiological signals to predict malignant VA and SCD, and to explore the sources of heterogeneity between studies.

## Methods

This review was reported according to the Preferred Reporting Items for Systematic Reviews and Meta-Analyses (PRISMA). The study protocol was registered on the international prospective register of systematic reviews (PROSPERO, reference number: CRD42021283464). Below we formulated the research question according to use the PICOTS system as provided by the *CHecklist for critical Appraisal and data extraction for systematic Reviews of prediction Modelling Studies (CHARM)-checklist*.^19^^,^^20^

### Population

Subjects from whom electrophysiological signals were obtained for the purpose of predicting the occurrence of the outcome(s) of interest were included. Electrophysiological signals considered eligible were ECG, intracardiac device recorded electrograms (EGM), holter-ECG, signal-averaged ECGs (SAECG), cardiac stress test ECG, and electrophysiological studies. Studies investigating participants <18 years old were excluded, no other criteria regarding eligibility of the population were applied.

### Index model

Supervised or semi-supervised ML or a DL model used to predict the outcome of interest, or any combinations thereof, were eligible. Studies were included regardless of the type of prediction model according to the checklist for CHARMS-checklist (i.e. development studies with and without external validation, external model validation with or without model updating).^19^ Studies were included only if electrophysiological signals were used as sole or primary model input.

### Outcome(s)

The outcome of interest was one (or a combination) of the following outcomes: (sustained) ventricular tachycardia (VT), ventricular fibrillation (VF), sudden cardiac death (SCD), in-hospital (IHCA) or out-of-hospital cardiac arrest (OHCA), or appropriate ICD therapy (shock or antitachycardia-pacing (ATP). Binary and time-to-event outcomes were considered both eligible.

### Timing and setting

The timing of predictions was at the moment of obtaining the electrophysiological signal, all prediction horizons were eligible. There were no restrictions on the setting the model was developed or validated in.

### Literature search

The MEDLINE (Ovid), EMBASE (Ovid), Scopus, Web of Science and Cochrane Library Databases electronic databases were systematically searched to identify studies published before September 2021. Databases were searched on September 1st 2021 using the following terms: ‘implantable cardioverter defibrillator’, ‘sudden cardiac death’, ‘machine learning’ and ‘electrocardiography’. The full search strategy is provided in the supplementary material (Supplementary Tables S1–S5). Such strategy, including terms and limits, was designed in collaboration with a medical information specialist. The reference lists of relevant papers were hand-searched to identify studies potentially missed by the electronic search.

### Study selection

The results from the electronic searches were imported into a reference management software and de-duplicated. Two review authors (M.K, B.D) conducted screening of studies independently with disagreements resolved through discussion or arbitration of a third reviewer (F.T).

### Risk of bias (quality) assessment

The risk of bias was assessed using *PROBAST: A Tool to Assess the Risk of Bias and Applicability of Prediction Model Studies*.^21^ All studies were scored on risk of bias for four categories (i.e. participants, predictors, outcome, and analysis). Low overall risk of bias was assigned if each domain was scored as low risk. High overall risk of bias was assigned if at least one domain was judged to be high risk of bias. Unclear overall risk of bias was assigned if at least one domain was judged unclear, and all other domains as low. The risk of bias assessment was performed independently by two authors (M.K, B.D). In cases of disagreement, both authors attempted to reach consensus. If no consensus was reached, a third reviewer was consulted to settle the disagreement (F.T).

### Synthesis of results

General study characteristics, study population and baseline characteristics (including sex distribution), type of electrophysiological signals used and analytical methods (i.e. model selection, feature selection, validation techniques) were extracted. Second, we extracted study estimates of sensitivity, specificity, positive predictive value, negative predictive value, accuracy, contingency tables and c-statistic (area under the curve). If studies reported insufficient details to reconstruct contingency tables, the respective authors were contacted to provide the missing data. Data extraction was performed by two independent reviewers (M.K, B.D). Studies were classified based on the database(s) used for model development in order to avoid overlap between studies that results from the use of publicly available datasets by multiple studies, and to reduce the potential for optimistically biased pooled performance estimates based on unrepresentative datasets. Databases that were classified as '*ad-hoc*' met the following criteria:

- The dataset was publicly available and may have been made available for challenges (e.g. the PhysioNet ECG challenge^22^);
- The dataset was developed with the primary aim for cooperative analysis and the development and evaluation of proposed new algorithms;
- The dataset may have been used as data source for multiple individual studies with similar research questions, leading to overlapping study populations;
- The dataset was considered unrepresentative (i.e. the dataset has an imbalanced outcome of interest that does not reflect a clinical setting, the datasets consists of outdated data, there is insufficient information on the origin of the data or population characteristics)

### Statistics

Exploratory meta-analysis was performed to reflect on and explain variations in the predictive performances of ML and DL models.^23^ Models were included in the meta-analysis if sufficient information was provided to reconstruct contingency tables consisting of true positive, false positive, true negative, and false negative results based on the specificity, sensitivity, prevalence and sample size. Pooled estimates of the diagnostic odds ratio (DOR) and the area under the summary receiver operator curve (AUROC) were calculated, the sensitivity and specificity were not pooled due to their dependency on the probability threshold. The DOR describes the odds of a positive prediction in those with the outcome relative to the odds of a positive prediction in those without the outcome. Summary receiver operator characteristic (ROC) curves were constructed based on a bivariate regression approach.^24^ Using parametric bootstrapping, the 95% confidence intervals around the AUROC were calculated.^25^ Pooled estimates of the predictive performance were calculated separately for models developed on an *ad-hoc* dataset (or a combination of *ad-hoc* datasets), taking into account the distinct differences in representativeness of these datasets. To reduce the risk of overlapping populations from *ad-hoc* databases between studies, the best performing model for each unique sample of subjects was selected and used to calculate pooled estimates. The I^2^ statistic was calculated to quantify the amount of inconsistency between studies. In cases of high heterogeneity, a series of sensitivity analyses were performed to explore potential sources of heterogeneity. First, we employed a leave-one-out approach in which we excluded one study at a time, to ensure that the results were not simply due to one large study or a study with an extreme result. Second, subgroup analyses were performed to examine whether the pooled accuracy of models varied by risk of bias, sample size, region of origin and the *ad-hoc* dataset that was used. Publication bias was visualised using funnel plots, Egger's test was used to test for publication bias. The trim and fill method proposed by Duval and Tweedie was used to estimate the number of studies missing from a meta-analysis and compute the summary estimate based on the complete data.^26^ A *P*-value of less than 0.05 was considered to be statistically significant. R software, version 3.6.2 (R Core Team) was used to analyse the pooled result, specifically the Meta-Analysis of Diagnostic Accuracy and the General Package for Meta-Analysis libraries.^27^, ^28^, ^29^

### Ethics

This meta-analysis study is exempt from ethics approval as data was collected and synthesised from previous studies.

### Role of the funding source

The funding source had no role in the study design, data collection, data analyses, interpretation, or writing of report.

## Results

**Fig. 1:**
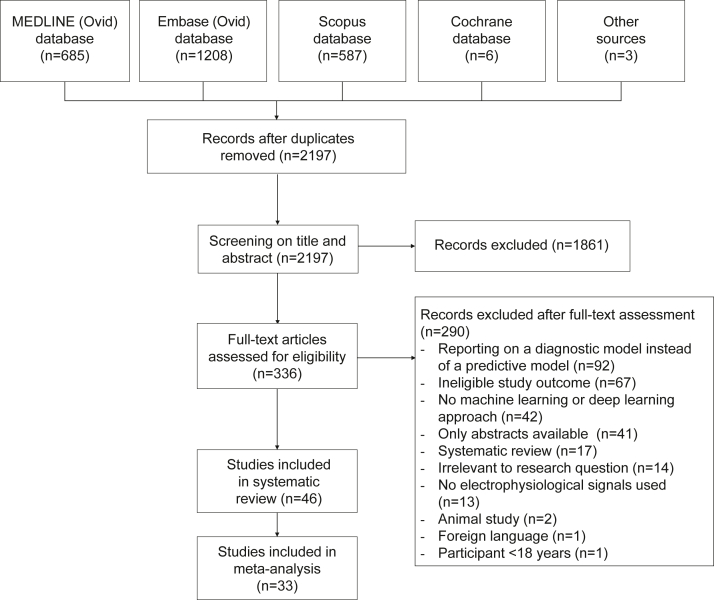
Study selection flow chart showing the results in each step of the systematic search to identify studies.

A total of 2486 studies were identified through the MEDLINE (n = 685), EMBASE (n = 1208), Scopus (n = 587) and Cochrane (n = 6) databases searches. Another three studies were identified through scrutiny of reference lists of relevant studies. After deduplication, a total of 2197 studies remained. Fig. 1 displays a flow diagram of the study selection process. Frequent reasons for exclusion were: reporting on a diagnostic model instead of a predictive model (n = 92), ineligible study outcome (n = 67) and no ML or DL approach (n = 42). Ultimately, a total of 46 studies were included in this review.^15^, ^16^, ^17^, ^18^^,^^30^, ^31^, ^32^, ^33^, ^34^, ^35^, ^36^, ^37^, ^38^, ^39^, ^40^, ^41^, ^42^, ^43^, ^44^, ^45^, ^46^, ^47^, ^48^, ^49^, ^50^, ^51^, ^52^, ^53^, ^54^, ^55^, ^56^, ^57^, ^58^, ^59^, ^60^, ^61^, ^62^, ^63^, ^64^, ^65^, ^66^, ^67^, ^68^, ^69^, ^70^, ^71^ Out of these 46 studies, 36 used one or more *ad-hoc* dataset(s) and were pooled in separate meta-analysis.^30^, ^31^, ^32^, ^33^, ^34^, ^35^, ^36^, ^37^^,^^39^, ^40^, ^41^, ^42^, ^43^, ^44^^,^^46^, ^47^, ^48^, ^49^, ^50^, ^51^, ^52^^,^^54^^,^^55^^,^^57^, ^58^, ^59^, ^60^, ^61^^,^^63^^,^^65^, ^66^, ^67^, ^68^, ^69^, ^70^, ^71^

### Machine learning and deep learning models developed on clinically-defined datasets

The characteristics of studies are summarised in Table 1, details on the electrophysiological signals used are displayed in Supplementary Material Table S6.^15^, ^16^, ^17^, ^18^^,^^38^^,^^45^^,^^53^^,^^56^^,^^62^^,^^64^ Two studies used intracardiac EGMs,^15^^,^^56^ seven used body surface ECG recordings^16^^,^^17^^,^^38^^,^^45^^,^^53^^,^^62^^,^^64^ and one study used ventricular monophasic action potentials (MAP) as model input.^18^ ECGs ranged from 10 s till 24 h in duration and differed in number of leads (1-, 3-, 7- and 12-leads) and sampling rate (125 Hz–1600 Hz). Support vector machine classifiers were implemented as prediction model in six studies,^15^^,^^17^^,^^18^^,^^56^^,^^62^^,^^64^ ensemble learning methods (random forests, decision tree) in three studies^15^^,^^38^^,^^45^ and artificial neural network in one study.^53^ Kwon et al. and Rogers et al. applied a deep learning model based on a convolutional neural network (CNN).^16^^,^^18^ Six studies developed a ML model for short-term prediction (horizons within a range 1 min till 72 h before event), the other four studies used a baseline recording as input to predict the event during a follow-up period that ranged from 21 till 44 months (i.e. long-term prediction). K-fold cross-validation and leave-one-out-cross validation were used for model validation in four studies validation,^17^^,^^56^^,^^62^^,^^64^ whereas a hold-out test set was used in six studies.^15^^,^^16^^,^^18^^,^^38^^,^^45^^,^^53^ External validation of the model was performed in two studies.^16^^,^^56^

**Table 1:** Study characteristics and predictive performance of studies included studies reporting on a machine learning and deep learning model.

Study characteristics				ML and DL modelling			Performance						Validation	
Author	n	Subjects	Study design	Endpoint (Prevalence)	Features	Algorithm	Sensitivity	Specificity	PPV	NPV	AUC	Accuracy	Internal validation	External validation
Au-Yeung et al.^15^	788	Prophylactic ICD recipient (77.3% male)	RCT	Appropriate ICD shock (3.3%)	HRV (non-linear domain, frequency-domain)	SVM and RF∗	74.0	74.0	N/A	N/A	81.0	N/A	80% training, 20% test	N/A
Do et al.^38^	1874	Hospitalised (66.9% male)	Retrospective, case–control study	IHCA (5.1%)	Trend analysis (slope, change)	RF∗, LR	94.6	63.2	N/A	N/A	82.9	N/A	80% train, 20% test	N/A
Lee et al.^53^	82 (104 recordings)	Hospitalised (sex distribution unknown)	Prospective cohort	VT (50%)	HRV (time-domain, non-linear Poincare, frequency-domain)	ANN	70.6	76.5	75.0	72.2	75.0	N/A	60% train, 40% test	N/A
Kwon et al.^16^	25 672	Hospitalised (53.1% male)	Retrospective cohort	IHCA (2.07%)	N/A	CNN	77.8	92.0	76.0	99.8	94.8	N/A	70% train, 30% test	Yes (n = 10,728)
Gleeson et al.^45^	295	Prophylactic ICD (74.2% male)	Retrospective cohort	ICD implantation or mortality (16.6%)	Spatial ECG parameters, complexity parameters and conventional ECG parameters	DT	N/A	N/A	N/A	N/A	75.0	N/A	60%, 40% test	N/A
Martinez-Alanis et al.^56^	91	ICD carriers (93.4% male)	Prospective cohort study	SCD (50%)	HRV (frequency and time-domain) and Heartprint Indices	SVM	N/A	N/A	N/A	N/A	68.0	67.65	10-fold CV	Yes
Ong et al.^17^	925	ED admissions (61.9% male)	Prospective cohort study	IHCA (4.6%)	HRV (time-domain, frequency-domain, and geometric parameters.)	SVM	81.4	72.3	12.5	98.8	78.1	N/A	LOOCV	N/A
Ramirez et al.^62^	597	CHF (71.2% male)	Prospective cohort study	SCD (8.2%)	ECG risk makers (repolarisation dispersion, TWA, HRT)	SVM	18.0	79.0	N/A	N/A	N/A	N/A	5-fold CV	N/A
Rodriguez et al.^64^	91	Idiopathic dilated cardiomyopathy (sex distribution unknown)	Prospective cohort study	VT/VF or SCD (15.4%)	HRV (time-domain, frequency-domain and non-linear Poincaré)	SVM	92.9	98.0	N/A	N/A	95.0	96.8	LOOCV	N/A
Rogers et al.^18^	42	Ischaemic cardiomyopathy (97.8% male)	Prospective cohort study	VT/VF (30.9%)	Mathematical timeserie features	SVM∗, CNN	84.6	86.2	73.3	92.6	90.0	85.7	70% training, 30% testing	N/A

ANN = artificial neural network, AUC = area under the curve, CNN = convolutional neural network, CHF = congestive heart failure, CV = cross validation, DT = decision tree, ECG = electrocardiography, ED = emergency department, RF = random forest, LOOCV = leave-one-out cross validation, LR = logistic regression, HRT = heart rate turbulence, HRV = heart rate variability, IHCA = in-hospital cardiac arrest, ICD = implantable cardioverter defibrillator, LOOCV = leave-one-out cross validation, N/A = not applicable, NPV = negative predictive value, PPV = positive predictive value, RCT = randomised controlled trial, SCD = sudden cardiac death, SVM = support vector machine, TWA = T-wave alternans, VT = ventricular tachycardia, VF = ventricular fibrillation.

**Fig. 2:**
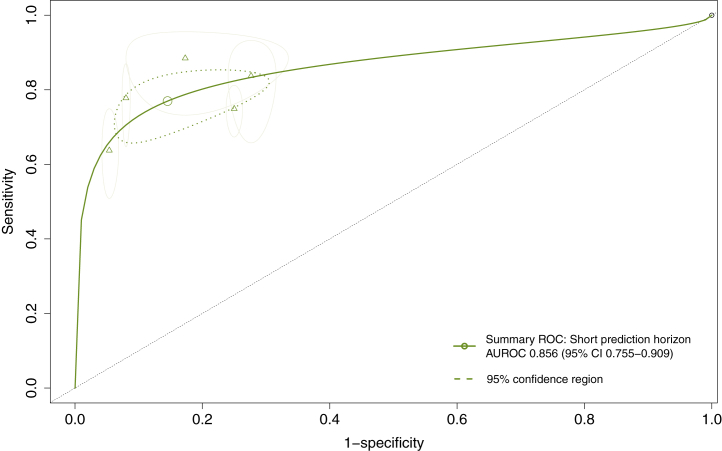
Summary ROC curves of five models developed to predict SCD or malignant VA on a short prediction horizon (time-to-event within 72 h). Point estimates are displayed for each individual study.

**Fig. 3:**
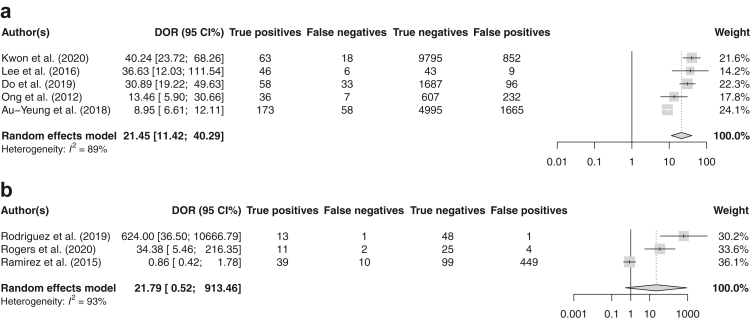
(a) Forest model of the diagnostic odds ratio (DOR) and 95% confidence intervals of models developed to predict on a short horizon (time-to-event within 72 h). (b) Forest model of the diagnostic odds ratio (DOR) and 95% confidence intervals of models developed to predict on a long horizon (time-to-event up to years).

Meta-analysis was performed for eight studies,^15^, ^16^, ^17^, ^18^^,^^38^^,^^53^^,^^62^^,^^64^ two studies did not report sufficient information regarding the predictive performance of the model to be able to reconstruct contingency tables.^45^^,^^56^ The sensitivity and specificity of these models ranged between 0.647-0.929 and 0.181–0.980, respectively (Supplementary Material Figs. S3 and S4). Prediction horizons differed substantially between individual studies, ranging from a time-to-event of minutes to hours (i.e. short-term) to a time-to-event of months to years (i.e. long-term). The pooled performance of five models (20,479 patients) developed for short-term prediction demonstrated a DOR of 21.45 (95% CI 11.42–40.29) and a summary AUROC of 0.856 (95% CI 0.755–0.909), with high heterogeneity (I^2^ = 89%) between studies (Fig. 2, Fig. 3a). Subgroup analyses for low vs. high risk of bias and sample size <500 vs. ≥500 subjects are displayed in the Supplementary Material Figs. S5 and S6. Leave-one-out sensitivity analysis showed each individual study to significantly affect the pooled estimate of the DOR (P < 0.05) (Supplementary Fig. S7). Three studies reported on a model developed to predict on a median time-to-event of 28–44 months (cumulative 702 patients), with a pooled DOR of 21.79 (95% CI 0.52–9.13.46, I^2^ = 93%) and a summary AUROC of 0.876 (95% CI 0.642–0.980). No sensitivity analyses were performed to explain heterogeneity considering the low number of studies.

Funnel plots for publication bias were visualised and are displayed in Supplementary Material Fig. S8, Egger's tests showed no evidence of publication bias. The trim-and-fill method identified two additional missing studies for short-term prediction that resulted in a pooled DOR of 13.99 (95 CI% 6.85–28.54), which is lower compared to the original analysis. Considering the low number of studies (k < 10), this assessment may not be reliable.

### Machine learning and deep learning models developed on ad-hoc datasets

The characteristics of ML and DL models developed using an *ad-hoc* dataset are summarised in Table 2. A total of 36 studies have been included, derived from eight different *ad-hoc* datasets. Detailed descriptions of the *ad-hoc* datasets are displayed in Table 3. The MIT-BIH SCD Holter database (SCDH) and the normal sinus rhythm database (NSRBD) were used in 27 and 28 studies, respectively.^22^ The SCDH database consists of 23 24-h ECG recordings of patients who suffered a sustained ventricular tachyarrhythmia (20 patients with VF, 3 with VT). Other open dataset used were the Creighton University ventricular tachyarrhythmia database (CUDB, 6 studies),^73^ Spontaneous Ventricular Tachyarrhythmia Database (MVTDB, 2 studies),^22^ AHA Database for Evaluation of Ventricular Arrhythmia Detectors (AHADB, 2 studies),^22^ the Fantasia database (1 study),^22^ Malignant Ventricular Arrhythmia Database (VFDB, 1 study)^75^ and the Paroxysmal Atrial Fibrillation Prediction challenge Database (PAFDB, 1 study).^74^ Supplementary Table S7 summarises the electrophysiological features that were used as input to the prediction models. Most commonly, studies used heart rate variability as model input, in particular (a combination of) features extracted the time-domain, frequency-domain, time-domain, time-frequency-domain and non-linear features. Other ECG features were related to the ECG morphology, such as intervals and amplitude of the QRS complex and ventricular repolarisation features (e.g. T-wave alternans). None of the studies reported on the external validation of a prediction model.

**Table 2:** Study characteristics and predictive performance of studies reporting on a prediction model developed on one or more *ad-hoc* datasets.

Study characteristics			ML and DL modelling			Performance						Validation	
Authors	No. cases/controls	Database	Algorithm	Features	Prediction interval	Sensitivity	Specificity	PPV	NPV	AUC	Accuracy	Internal	External
Acharya et al.^31^	20 SCD/18 controls	SCDH; NSRD	DT, KNN, SVM∗	ECG (DWT decomposition, non-linear feature extraction: FD, entropy)	2 min	100.0	97.2	97.5	100.0	N/A	98.7	10-fold CV	N/A
Acharya et al.^30^	20 SCD/18 controls	SCDH; NSRD	KNN∗, PNN, SVM, DT	HRV (RQA, non-linear)	4 min	94.4	80.0	N/A	N/A	N/A	86.8	10-fold CV	N/A
Alfarhan et al.^32^	20 SCD/20 controls	SCDH; NSRD	KNN∗ and LDA	HRV (frequency-domain, time-domain and QRS complex features and VR features)	10 min	N/A	N/A	N/A	N/A	N/A	97.0	10-fold CV	N/A
Amezquita-Sanchez et al.^33^	23 SCD/18 controls	SCDH; NSRD	Enhanced probabilistic NN	WPT to decompose data in frequency bands, non-linear feature extraction (homogeneity index)	20 min	N/A	N/A	N/A	N/A	N/A	95.8	50% train, 20% validation, 30% test	N/A
Bayasi et al.^34^	16 VA/18 controls	NSRD; SCDH; CUDB; AHADB	LDA	Segments (PQ, PS, RT, QP, SP)	3 h	98.9	N/A	98.4	N/A	99.9	99.1	10-fold CV	N/A
Calderon et al.^35^	16 SCD/20 controls	SCDH and Fantasia database	DT, KNN, SVM, LR and ANN∗	Segments (PS,Q T, ST, PR and RR)	2 min	91.0	93.0	N/A	N/A	N/A	92.0	8-fold CV	N/A
Cappielo et al.^36^	32 VA/32 controls	CUDB; PTBDB	Hybrid prediction index	Phase-space portraits characteristics	354 ECG beats	96.9	100.0	100.0	97.0	N/A	98.4	LOOCV	N/A
Devi et al.^37^	18 SCD/18 controls/15 CHF	SCDH; BIDMC Congestive Heart Failure, NSRD	DT, KNN∗, SVM	HRV (CWT, time-domain, frequency-domain, time-frequency and non-linear)	10 min	75.0	87.5	75.0	75.0	N/A	83.33	75% train, 25% test	N/A
Ebrahimzadeh et al.^39^	35 SCD/35 normal)	SCDH; NSRD	MLP, KNN, SVM, ME classifier∗	HRV (time-domain, frequency-domain, time-frequency, non-linear)	13 min	82.2	85.7	83.3	85.3	N/A	82.9	train 70%, test 30%	N/A
Ebrahimzadeh et al.^40^	23 SCD/18 controls	SCDH; NSRD	MLP	HRV (time-domain, frequency-domain, time-frequency, non-linear)	12 min	82.7	85.1	84.7	83.1	N/A	83.9	LOOCV	N/A
Ebrahimzadeh et al.^42^	35 SCD/35 normal	SCDH; NSRD	MLP∗ and KNN	HRV (time-domain, frequency-domain, time-frequency and non-linear (DFA, Poincaré))	4 min	83.8	16.0	84.0	83.8	N/A	83.9	LOOCV	N/A
Ebrahimzadeh et al.^41^	35 SCD/35 controls	SCDH; NSRD	ANN	HRV (time-domain, frequency-domain, time-frequency-domain)	2 min	N/A	N/A	N/A	N/A	N/A	91.23	LOOCV	N/A
Fairooz et al.^43^	18 SCD/18 normal	SCDH; NSRD	SVM	CWT transformation, subsequent feature extraction (intervals, amplitudes, TWA)	30 min	100.0	100.0	100	100	N/A	100	Train 77.78%, test 22.22%	N/A
Fujita et al.^44^	20 SCD/18 normal	SCDH; NSRD	SVM∗, DT and KNN	HRV (DWT, non-linear features: Renyi entropy, fuzzy entropy, Hjorths parameters and Tsallis entropy)	4 min	95.0	94.4	95.0	94.4	N/A	94.7	10-fold CV	N/A
Houshyarifar et al.^47^	23 SCD/36 normal	SCDH; NSRD	KNN, SVM∗	HRV (non-linear, spectrum HOS features and time-domain)	4 min	N/A	N/A	N/A	N/A	N/A	94.5	10-fold CV	N/A
Houshyarifar et al.^46^	23 SCD/36 normal	SCDH; NSRD	KNN, SVM∗	HRV (non-linear recurrence and Poincaré plot)	4 min	84.25	96.8	N/A	N/A	N/A	93.3	10-fold CV	N/A
Jeong et al.^48^	58 VF/60 controls	CUDB, MVTDB, PAFDB and NSRDB	ANN	HRV (time-domain and non-linear Poincare)	80 s	N/A	N/A	N/A	N/A	N/A	88.18	10-fold CV	N/A
Joo et al.^49^	78 ICD patients	MVTB	ANN (VF)	HRV (time-domain, frequency-domain and non-linear Poincaré)	5 min	88.9	92.9	72.7	97.5	N/A	92.9	66% train 33% test	N/A
Khazaei et al.^50^	23 SCD/18 controls	SCDH; NSRD	DT∗, KNN, NB and SVM	HRV (non-linear: RQA and increment entropy)	6 min	95.0	95.0	N/A	N/A	N/A	95.0	10-fold CV	N/A
Lai et al.^52^	18 SCD/18 controls	SCDH; NSRD	KNN∗, DT, NB	Ventricular repolarisation features	60 min	99.5	98.3	98.3	N/A	N/A	98.9	5-fold CV	N/A,
Lai et al.^51^	28 SCD/18 controls	AHADB; SCDH; NSRD	KNN, DT, NB, SVM and RF∗	Ventricular repolarisation features∗	30 min	99.8	99.0	99.4	99.6	N/A	99.5	5-fold CV	N/A
Lopez-Caracheo et al.^54^	9 SCD/9 controls	SCDH; NSRD	HFD, BD, and KFD algorithms	HRV (Non-linear: Katz, Higuchi and Box Dimension)	14 min	N/A	N/A	N/A	N/A	N/A	91.4	50% train, 50% test	N/A,
Mandala et al.^55^	22 VA/18 controls	NSRD; VFDB	SVM, NB∗, DT	HRV (time-domain) and QRS complex features	25 min	93.3	86.7	N/A	N/A	N/A	N/A	5-fold CV	N/A
Mirhoseini et al.^57^	19 SCD/18 controls	SCDH; NSRD	SVM∗, DT	HRV (time-domain, frequency-domain, time-frequency, non-linear)	1 min	N/A	89.5	87.5	81.0	N/A	83.2	10-fold CV	N/A
Murugappan et al.^58^	18 SCD/18 controls	SCDH; NSRD	SVM,∗ subtractive fuzzy clustering, and neuro-fuzzy classifier	HRV (Non-linear features: Largest Lyapunov Exponent/approximate entropy/Sample entropy/Hurst exponent)	5 min	97.1	97.1	100.0	97.6	N/A	100.0	10-fold CV	N/A
Murugappan et al.^59^	20 SCD/18 controls	SCDH; NSRD (40 vs 36 holter)	KNN∗ and fuzzy classifier	HRV (time-domain)	5 min	92.2	95.3	95.4	N/A	N/A	93.71	10-fold CV	N/A
Murukesan et al.^60^	23 SCD/18 controls	SCDH; NSRD	SVM∗, PNN	DWT and HRV feature extraction (time-domain, frequency-domain, time-frequency, non-linear)	2 min	93.3	100.0	N/A	N/A	N/A	96.4	train 70% test 30%	N/A
Parsi et al.^61^	78 ICD carriers	MVTB (135 pre VT/126 controls)	SVM, RF and KNN∗	HRV (time and frequency-domain, HOS features, non-linear Poincaré)	5 min	88.8	94.2	N/A	N/A	N/A	91.5	LOOCV	N/A
Riasi et al.^63^	40 VT/40 controls	SCDH; NSRD and CUDB	SVM	Morphological features (area under ascending/descending/total T-wave and R-wave, beat to beat correlations, intervals)	20 s	88.0	100.0	N/A	N/A	N/A	94.0	75% train 25% test	N/A
Shi et al.^65^	20 SCD/18 controls	SCDH; NSRD	KNN	HRV (EMD for entropy parameters, time-domain and frequency-domain)	14 min	97.5	94.4	N/A	N/A	N/A	96.1	10-fold CV	N/A
Shen et al.^69^	23 SCD/20 controls	SCDH and database	LSM∗, DBNN, BPNN	HRV (FFT and frequency-domain)	2 min	75.0	N/A	N/A	N/A	N/A	87.5	46% train, 56% test	N/A
Taye et al.^66^	78 ICD carriers	MVTDB (135 pre VT/126 controls)	1-D CNN	N/A	60 s	83.2	86.4	N/A	N/A	78.0	84.6	10-fold CV	N/A
Taye et al.^67^	27 VF/28 controls	CUDB, PAFDB, NSRDB	Fully connected ANN	HRV (time-domain, frequency-domain, non-linear Poincare), QRS complex features	30 s	98.4	99.0	N/A	N/A	99.0	98.6	10-fold CV	N/A
Tseng et al.^68^	81	CUDB	2D CNN, 2D-STFT	N/A	5 min	98.0	N/A	N/A	N/A	N/A	88.0	80% train and 20% validation	Two real cases as validation
Tsjui et al.^70^	20 SCD/20 controls	Not specified	R-LLGMn	HRV (time-domain)	5 min	N/A	N/A	N/A	N/A	90.0	82.5	LOOCV	N/A
Vargas-Lopez et al.^71^	23 SCD/18 controls	SCDH; NSRD	MLP	EMD, subsequent entropy and fractal dimension feature extraction	25 min	N/A	N/A	N/A	N/A	N/A	94.0	45% and 55% validation	N/A,

AHADB = AHA Database for Evaluation of Ventricular Arrhythmia Detectors, ANN = artificial neural network, AUC = area under the curve, BPNN = back-propagation neural network, CNN = convolutional neural network, CUDB=Creighton University ventricular tachyarrhythmia database, CV = cross validation, CWT=Continuous Wavelet Transform, DFA = detrended fluctuation analysis, DWT = Discrete wavelet transform, DBNN = decision-based neural network, DT = Decision Tree, ECG = electrocardiography, EMD = empirical mode decomposition, EMG = intracardiac electrogram, FD= Fractal Dimension, FFT = fast Fourier transform, HOS = higher order spectral, HRV = heart rate variability, KNN = k-nearest neighbour, LMS = least mean square, MVTDB = Spontaneous Ventricular Tachyarrhythmia Database, MLP = multi-layer perceptron, ME = maximum entropy, NSRBD = normal sinus rhythm database, PPV = positive predictive value, PAFDB = paroxysmal atrial fibrillation prediction challenge database, PNN = probabilistic neural network, LOOCV = leave-one-out cross validation, LDA = linear discriminant analysis, NB = naïve Bayes, NPV = negative predictive value, RF = random forest, R-LLGMn = recurrent log-linearised Gaussian mixture network, SCD = sudden cardiac death, SCDH = MIT-BIH SCD Holter database, SVM = support vector machine, RQA = recurrence quantification analysis, VFDB = Malignant Ventricular Arrhythmia Database, VF = ventricular fibrillation, VT = ventricular tachycardia, VR=Ventricular repolarisation, WPT = wavelet packet transform, 2D-STFT = two-dimensional short-time Fourier transform.

**Table 3:** Characteristics of the *ad-hoc* datasets used for the prediction of sudden cardiac death or malignant ventricular arrhythmias.

Name	Subjects included in the database	No. recordings	Type	Frequency
Massachusetts Institute of Technology-Beth Israel Hospital SCD Holter database (SCDH)^22^	Recordings of subjects before SCD or sustained VT onset as well as a few seconds later. 18 subjects (8 female, 13 female, 2 unknown) had underlying sinus rhythm (4 with intermittent pacing), 1 subject was continuously paced, and 4 subjects were diagnosed with atrial fibrillation. All subjects had a sustained ventricular and most had an actual cardiac arrest.	23 (20 subjects with VF) subjects with 46 recordings (lead I and lead II for each subject)	24-h ECG	250 Hz
Massachusetts Institute of Technology-Beth Israel Hospital normal sinus rhythm database (NSRBD)^22^	Subjects (5 male, 13 female) included in this database were found to have had no significant arrhythmias. Ages between 20 and 50 years old.	18 subjects with 36 recordings (lead I and lead II for each subject)	24-h ECG	128 Hz
Malignant Ventricular Arrhythmia Database (VFDB)^72^	Subjects who experienced episodes of sustained ventricular tachycardia, ventricular flutter, and ventricular fibrillation. No details on subject's sex.	22 subjects with 22 recordings	30-min ECG	250 Hz
Creighton University ventricular tachyarrhythmia database (CUDB)^22^^,^^73^	Subjects who experienced episodes of sustained ventricular tachycardia, ventricular flutter, and ventricular fibrillation, 5 records were from paced subjects. No details on subject's sex.	35 subjects with 35 recordings	8-min ECG	250 Hz
AHA Database for Evaluation of Ventricular Arrhythmia Detectors (AHADB)^22^	Subjects with no ventricular ectopy, isolated unifocal PVCs, isolated multifocal PVCs, ventricular bi- and trigemini, R-on-T PVCs, ventricular couplets, ventricular tachycardia, ventricular flutter/fibrillation. No details on subject's sex.	80 subjects with 80 two-lead recordings	3-h ECG (2-channel)	250 Hz
Paroxysmal atrial fibrillation prediction challenge database (PAFDB)^74^	Subjects who have paroxysmal atrial fibrillation and subjects with no documented AF. No details on subject's sex.	48 subjects with 50 recordings	30-min ECG	128 Hz
Spontaneous Ventricular Tachyarrhythmia Database (MVTDB)^22^	Subjects with an ICD who experienced an episode of ventricular tachycardia or ventricular fibrillation. No details on subject's sex.	78 subjects with 135 pairs of RR intervals	EGMs	1000 Hz
Fantasia^22^	Twenty young (21–34 years old) and twenty elderly (68–85 years old) healthy subjects underwent 120 min of continuous supine resting while continuous ECG (20 male, 20 female subjects).	40 individuals with 40 recordings	120-min ECG	250 Hz

ECG = electrocardiography, EGM = intracardiac electrogram, PVC = premature ventricular complex, SCD = sudden cardiac death, VF = ventricular fibrillation.

**Fig. 4:**
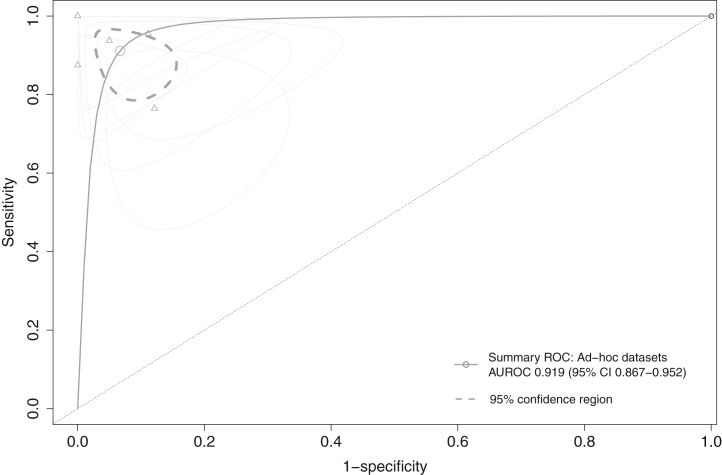
Summary ROC curves of best performing models developed per (combination of) *ad-hoc* dataset.

**Fig. 5:**
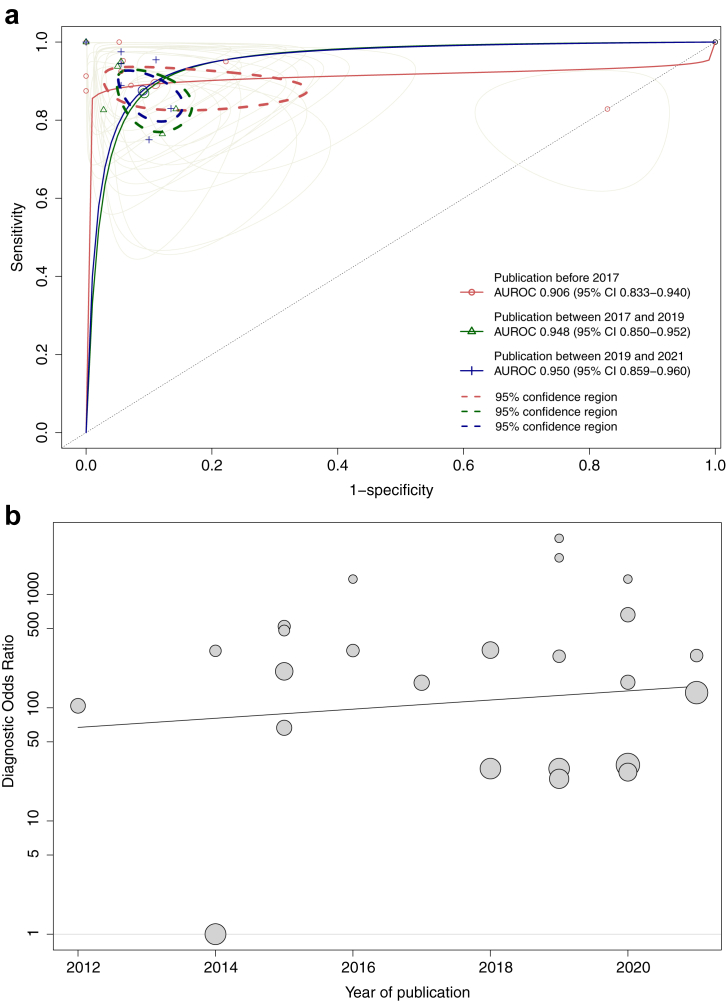
(a) Summary ROC curves of models developed on *ad-hoc* datasets per over the course of time. (b) Diagnostic odds ratio of models developed using *ad-hoc* datasets between 2012 and August 2021.

Overall, 24 studies (344 unique patients) that reported on models developed using *ad-hoc* datasets provided sufficient information for meta-analysis of pooled data. The sensitivity and specificity of these models ranged between 0.750-1.000 and 0.171–1.000, respectively (Supplementary Fig. S9). Predictions horizons ranged from a time-to-event of 20 s until 3 h. The pooled DOR of the seven best performing models for each of the (combination of) datasets was 282.04 (95% CI 62.96–1263.40) and the summary AUROC was 0.919 (95% CI 0.867–0.952) (Fig. 4). Heterogeneity was moderate (I^2^ = 49%) with all studies significantly changing the pooled DOR if excluded in sensitivity analyses (*P* < 0.05) (leave-on-out sensitivity analysis is shown in Supplementary Material Fig. S15). The pooled summary AUROCs per time period of publication were 0.906 (95% CI 0.833–0.940), 0.948 (95% CI 0.850–0.952) and 0.950 (95% CI 0.859–0.960), for studies published before 2017, between 2017 and 2019 and studies between 2020 and 2021, respectively (Fig. 5a). The DOR over time per study is displayed in Fig. 5b. All sensitivity analysis are displayed in the Supplementary Material Figs. S11–S14 and S17 visualises the DORs per ML or DL algorithm that was used. The funnel plot for publication bias is visualised in Supplementary Material Fig. S16. Egger's test showed evidence of publication bias in favour of studies reporting higher DOR (*P* = 0.003). The trim-and-fill method indicated four potential missing studies and estimated a DOR of 66.97 (95% CI 13.21–339.58), which is substantially reduced compared to the original analysis.

### Risk of bias assessment

The risk of bias assessment is presented in Supplementary Figs. S1 and S2. The studies that reported on a model developed using clinically-defined data were scored as low (4 studies), high (2 studies) and unclear risk of bias (4 studies). In studies that reported on model development using *ad-hoc* datasets, 5 studies were scored as low risk, 24 as high risk and 7 as unclear risk.

## Discussion

We systematically identified and summarised ML and DL models that used electrophysiological signals to predict malignant VA and SCD, and conducted exploratory meta-analyses to explain the sources of heterogeneity. AI has the potential to extract and process features from high dimensional complex electrophysiological signals and learn complex, hidden relationships between these features and the onset of malignant VA or SCD. Overall, ML and DL models showed high predictive performance, with models developed using (a combination of) *ad-hoc* datasets achieving particularly excellent performance with a summary AUROC of 0.919 (95% CI 0.867–0.952). On the other hand, studies were characterised by high risk of bias and considerable heterogeneity in terms of model performance, electrophysiological signals used, sample sizes and settings. In addition, very few studies have reported on the performance of a model when tested on an external patient cohort, which is crucial for assessing its generalisation ability. It is essential for these important methodological considerations to be addressed in future studies in order for AI models to be adopted in clinical practice.

### Current barriers to clinical implantation: external validation and model deployment

The majority of research activity in the field of VA prediction using ML and DL has been undertaken in a pre-clinical setting using *ad-hoc* datasets. In particular, two *ad-hoc* datasets (SCDH and NSRD) comprising a total of 41 patients have been exhaustively utilised for model development (respectively 27 and 28 studies). Publicly available datasets have stimulated progress in model development over the past decades, by ensuring quality control and circumventing barriers such as patient consent, quality control, costs and disparate data sources.^76^ Nevertheless, these *ad-hoc* datasets were limited in sample size and amount of electrophysiological signals, making the derived models vulnerable to overfitting. This may lead to overly optimistic estimates of model performance. Moreover, the robustness of these model may be jeopardised by the use of datasets that do not accurately represent the target population, leading to a model that is susceptible to approximate noise in the training data rather than underlying patterns of interest.^77^ Expanding current *ad-hoc* datasets through the inclusion of more subjects and electrophysiological signals, and subsequently conducting external validation of derived models is paramount for establishing the robustness, reproducibility and generalizability.^23^ Second, ML and DL models could serve distinct clinical purposes (e.g. early-warning system, risk stratification, screening tool for general population), and therefore require different integration within clinical workflows. However, in order for ML models to have a meaningful impact on clinical practice it is critical to integrate them into medical workflows so that their impact on patients and clinicians can be assessed. The *ECG AI-Guided Screening for Low Ejection Fraction (EAGLE)* trial was among the first to specifically evaluate the use of an AI-tool for screening of heart failure patients in an integrated, real-world workflow using ECG.^78^ The EAGLE trial demonstrated that use of the AI-ECG model increased the number of low LVEF diagnoses despite only a modest increase in the use of echocardiography was observed. At present, no trial has evaluated the impact of a ML-based model for the prediction of malignant VA or SCD in clinical practice. Finally, the impact of an ML or DL model on clinical practice is largely dependent on epidemiological factors such as the pre-test probability. For example, Au-Yeung et al. performed a secondary analysis of patients implanted with an ICD in the randomised-controlled SCD-HeFT trial, using HRV features extracted from EGMs for the prediction of appropriate ICD-therapy.^15^ Despite the reasonable AUROC of the developed models (AUROC = 0.81), this still led to a disproportional absolute number of false positive predictions with a prevalence of 3.3% of appropriate ICD therapy. In addition, the model developed by Kwon et al. for predicting in-hospital cardiac arrest (prevalence of 0.78%), resulted in 845 false positive predictions compared to 64 true positive predictions on an external dataset, despite having an AUROC of 0.948 and specificity of 92.2%.^16^ In other words, the clinical utility of ML models is limited if used in a low prevalence setting, unless they are designed to have very high specificity. This highlights the importance of considering the prevalence of the outcome being predicted when determining the clinical utility of a model.

### Explainability and model transparency

Models developed using ML and DL techniques are often criticised for their lack of explainability of the predictions they provide. The emerging field of explainable-AI is rapidly evolving and could aid in providing human-interpretable predictions. For example, Kwon et al. used the saliency method to visualise the ECG regions used by the model to predict IHCA, which showed model predictions predominantly based on QRS complex and the T-waves.^16^ However, this encourages us to probe the causal (pathophysiological) pathway, such as the presence of a fibrotic tissue or abnormalities in intracellular calcium homeostasis as the substrate for malignant VA onset.^79^ A pipeline for mechanistic underpinning of model predictions was constructed by Rogers et al., who used the morphology of individual ventricular MAPs in patients with an ischemic cardiomyopathy to predict malignant VA. Their findings showed that the arrhythmic risk was predicted by prolonged phase II repolarisation which potentially reflects abnormal calcium handling, providing clinicians with interpretable ML predictions. In addition, considering the dynamic and complex nature of malignant VA onset it is important for prediction models to take into account persistent substrate as well as transient triggers for arrhythmia onset. The potential of repeated electrophysiological recordings per patients instead of features measured once at baseline was assessed by Perez-Alday et al., who found differences in short-term and long-term predictive accuracy of ECG features for SCD.^80^ Leveraging ML techniques for survival predictions using time-varying covariates has the potential to capture triggers for malignant VA on top of baseline predictors.^81^

### Limitations

An important limitation to this systematic review was the high percentage of included studies that reported insufficient data to be added meta-analysis of included papers (14 studies reported insufficient data to calculate contingency tables for meta-analysis), which could have affected the pooled summary estimates. Given the exploratory nature of the meta-analysis the pooled estimates are provided primarily for reference, and should be considered as hypothesis-generating. Second, this study did not include conventional statistical methods which impedes comparisons between AI and statical approaches. Third, recent population wide autopsy data published by Tseng et al. illustrated that 40% of deaths attributed to stated SCD were not sudden or unexpected, and nearly half of presumed SCDs were not arrhythmic.^82^ The pooled results in this meta-analysis could be imprecise considering both SCD and malignant VA were eligible as prediction outcome.

### Conclusion

Machine learning and deep learning have a potential for personalised prediction of malignant ventricular arrhythmias and could provide clinicians with early warning-systems and risk-stratification tools. Despite a substantial number of studies using ML or DL models to predict malignant VA and SCD, studies were predominately conducted using small *ad-hoc* datasets, lacked an external validation and were in general characterised by high risk of bias. It is pivotal that future studies meet methodological standards, are derived from multi-centric clinical datasets that capture sufficient between-subject variation, and are integrated into clinical work-flows in parallel with conventional care to assess their reproducibility, generalisability and utility.

## Contributors

FT, BD, SR, SN, NB, RK, PC and MK contributed to the conception and design of the study. FT, BD and MK contributed to the literature search and data extraction. FT, BD, SR, SN, RK, AW, PC and MK contributed to data analysis and interpretation. FT, BD, SR, SN, NB, RK, AW, PC and MK contributed to critical revision of the manuscript. MK, FT, BD and SR accessed and verified the underlying data. FT, BD, SR, SN, RK, AW, NB, PC and MK contributed to writing the manuscript, and all authors approved the manuscript.

## Data sharing statement

All data for this systematic review and meta-analysis were obtained from published studies. Data extracted for this review will be made available upon a reasonable request. For access, please email the corresponding author. The database search strategies are provided as Supplementary Material.

## Declaration of interests

SN has Grants or contracts from 10.13039/100000002National Institutes of Health HL149134. AW has Grants or contracts from Dutch Heart Foundation (Predict2), consultancy fee from LQTtherapeutics and Cydan and participates on a Data Safety Monitoring Board or Advisory Board for the LEAP trial. RK, FT, MK, SR, BD, PC, NB have no conflict of interests.
