# Supplementary material for: Machine learning of electrophysiological signals for the prediction of ventricular arrhythmias: systematic review and examination of heterogeneity between studies
Source: eBioMedicine. 2023 Feb 9;89:104462. doi: 10.1016/j.ebiom.2023.104462 (PMC9945642; doi:10.1016/j.ebiom.2023.104462)
Supplement: Supplementary Figs. S1–S7 and Tables S1–S17 [file mmc1.docx]

**Supplementary material**

Supplementary Table 1. Medline (Ovid) database search

Supplementary Table 2. Embase (Ovid) database search

Supplementary Table 3. Scopus database search

Supplementary Table 4. Web of Science database search

Supplementary Table 5. Cochrane database search

Supplementary Table 6. Details regarding the electrophysiological signals used by models (n=10) developed on clinically-defined datasets

Supplementary Table 7. Features extracted from electrophysiological signals and used as input for the ML and DL models

Supplementary Figure 1. Risk of bias assessment of studies (n=10) developed using a clinically-defined dataset according to PROBAST: A Tool to Assess the Risk of Bias and Applicability of Prediction Model Studies

Supplementary Figure 2. Risk of bias assessment of studies (n=36) developed using ad-hoc datasets according to PROBAST: A Tool to Assess the Risk of Bias and Applicability of Prediction Model Studies

Supplementary Figure 3. Forest model of the specificity (Panel 3a) and sensitivity (Panel 3b), and the 95% confidence interval of models developed to predict on a short horizon (within 72 hours)

Supplementary Figure 4. Forest model of the specificity (Panel 4a) and sensitivity (Panel 4b), and the 95% confidence interval of models developed to predict on a long horizon

Supplementary Figure 5. Forest model of the diagnostic odds ratio (DOR) and 95% confidence interval for low and high risk of bias

Supplementary Figure 6. Forest model of the diagnostic odds ratio (DOR) and 95% confidence interval for sample sizes <500 and ≥500

Supplementary Figure 7. Leave-one-out sensitivity analysis of models developed to predict on a short horizon (Panel 7a) and long horizon (Panel 7b)

Supplementary Figure 8. Funnel plot for models developed to predict on a short horizon (Panel 8a), and combined short and long horizon (Panel 8b). Grey markers represent original studies, white markers represent filled studies.

Supplementary Figure 9. Forest model of the specificity (Panel 9a) and sensitivity (Panel 9b), and the 95% confidence interval of models developed using ad-hoc datasets

Supplementary Figure 10. Forest plot of diagnostic odds ratio (DOR) and 95% confidence interval for models developed using ad-hoc datasets

Supplementary Figure 11. Forest plot of diagnostic odds ratio (DOR) and 95% confidence interval for models developed using ad-hoc datasets per ad-hoc dataset or combination of datasets

Supplementary Figure 12. Forest plot of diagnostic odds ratio (DOR) and 95% confidence interval for models developed using ad-hoc datasets per year of publication

Supplementary Figure 13. Forest plot of diagnostic odds ratio (DOR) and 95% confidence interval for models developed using ad-hoc datasets per risk of bias

Supplementary Figure 14. Forest plot of diagnostic odds ratio (DOR) and 95% confidence interval for models developed using ad-hoc datasets per region of origin

Supplementary Figure 15. Leave-one-out sensitivity analysis of models developed using ad-hoc datasets

Supplementary Figure 16. Funnel plot for models developed using ad-hoc dataset. Grey markers represent original studies, white markers represent filled studies.

Supplementary Figure 17. Bubble plot of the diagnostic odds ratio (DOR) for models developed using ad-hoc databases per type of algorithm

| **Supplementary Table 1.** Medline (Ovid) database search | |  |
| --- | --- | --- |
| Database(s): Ovid MEDLINE(R) ALL 1946 to August 03, 2021 | |  |
| # | Searches | Results |
| 1 | exp Electrocardiography/ | 209920 |
| 2 | (electrocardiogra* or "ECG*" or X-ECG or CPX or EGM or "Holter ECG" or "single averaged ECG*" or SAECG or "12-Lead ECG*" or "twelve lead ECG*").ti,ab,kf. | 147272 |
| 3 | ((cardi* or heart*) adj3 ("electrogra*" or "electro-gra*" or "exercis* test" or "stress test")).ti,ab,kf. | 3123 |
| 4 | ("T-wave alternans" or TWA or MWTA or "Index of Cardiac Electrophysiological Balance" or iCEB or "Heart rate variability" or HRV or "Heart rate turbulence" or HRT or "TpTe-interval" or TpTe or "Tpeak-Tend" or Tpe or "Tp-e" or "QT-dispersion*" or QTd or "QRS-duration*" or QRSd or "QT-interval" or "QT-time" or "Fragmented QRS" or fQRS or "global electrical heterogeneity" or "ventricular gradient*" or "spatial* ventricular gradient*" or "Early Repolarisation" or "QT Dynamicity").ti,ab,kf. | 54871 |
| 5 | or/1-4 | 302355 |
| 6 | exp Artificial Intelligence/ or exp Machine Learning/ or exp Neural Networks, Computer/ or exp Computer Simulation/ or exp Pattern Recognition, Automated/ | 384675 |
| 7 | ((machine or comput* or artificial* or deep or automat* or reinforce* or feature) adj3 (model* or intelligence or reasoning* or rational* or learn* or detect* or interpret* or algorithm* or simulat* or rank* or detect*)).ti,ab,kf. | 234631 |
| 8 | ("decision network" or "support vector machine*" or "vector machine*" or "deep architec*" or "convolution* neural network*" or "comput* vision" or "comput* aid* diagnos*" or "ensemble learning" or "ensemble method*" or "random forest*" or "random decision forest*" or "random survival* forest*" or ResNet or DenseNEt or "convolution network" or ANN or perceptron or radial basis or "k-Nearest Neighbor" or "k-nn" or "multilayer perceptron" or "recurrent neural network" or "Long short-term memory" or LSTM).ti,ab,kf. | 70517 |
| 9 | ((auto* or auto encod* or autoencod*) adj3 (quantif* or assess* or interpret* or identificat* or detect* or classif* or clust*)).ti,ab,kf. | 49754 |
| 10 | ((unsupervis* or supervis* or hierarch* or automat* or algorit* or ensemble or transfer*) adj3 (cluster* or classif* or learning)).ti,ab,kf. | 62526 |
| 11 | ((neural or bayesian or "generative adversarial") adj3 (network* or learn*)).ti,ab,kf. | 74065 |
| 12 | or/6-11 | 647101 |
| 13 | 5 and 12 | 10143 |
| 14 | exp Death, Sudden, Cardiac/ or exp Arrhythmias, Cardiac/ or *Defibrillators, Implantable/ or exp Heart Arrest/ or exp Electric Countershock/ | 267863 |
| 15 | ((ventricular* or cardiac or heart) adj3 (arrhythmia* or fibrillation or tachycardia or tachyarrhythmia* or flutter*)).ti,ab,kf. | 83814 |
| 16 | (sudden adj3 ((cardiac or heart) adj3 (arrest* or death*))).ti,ab,kf. | 21741 |
| 17 | (asystol* or "Cardiac Resynchronization Therapy Devices" or "CRT-D" or "implant* cardioverter-defibrillator*" or ICD* or "ICD therap*" or "ICD shock*" or "defibrillator shock*" or defibrillation* or "antitachycardia pac*" or "device* discharg**" or "electric* countershock*" or cardioversion* or "shock* rhythm*").ti,ab,kf. | 68672 |
| 18 | or/14-17 | 339188 |
| 19 | 13 and 18 | 3063 |
| 20 | exp Prognosis/ or exp ROC Curve/ or exp "Predictive Value of Tests"/ or exp Risk Assessment/ or exp Incidence/ | 2334707 |
| 21 | ((predict* or risk* or prognos*) adj3 (recall or precision or outcome* or assess* or chart* or model* or test* or value* or variable* or score* or algorithm* or tool* or engine or equation* or calculat* or instrument* or method* or support* or rule*)).ti,ab,kf. | 850131 |
| 22 | (prognos* or predict* or "area under the curve*" or "ROC Curve" or incidence or "youden index" or "youden statistic*" or "C statistic").ti,ab,kf. | 3006922 |
| 23 | ep.fs. | 1854962 |
| 24 | exp Multicenter Study/ or exp observational study/ or exp cohort studies/ or exp longitudinal studies/ or exp follow-up studies/ or prospective studies/ or retrospective studies/ or (cohort* or longitudinal or prospective or retrospective or follow-up or follow up).ti,ab,kf. or (cohort adj3 (historic* or prospecti* or restrospec* or longitud* or follow-up or follow up or stud*)).ti,ab,kf. or exp validation study/ or ("validat* stud*" or observat* stud* or "replicat* stud*").ti,ab. or (cohort stud* or validation stud* or longitudinal stud* or prospec stud* or resrospective stud* or validation stud*).pt. | 3685821 |
| 25 | or/20-24 | 7444749 |
| 26 | 19 and 25 | 1346 |
| 27 | ((exp animals/ or exp veterinary medicine/ or animal*.jw.) not humans/) or (experimental model or animal* or monkey* or horse* or racehorse* or sheep or ?ovine or lamb* or goat* or pig* or swine* or porcine* or pup* or dog*or canine* or bitch* or beagle* or feline* or rodent* or rabbit* or rat* or mice* or mouse or murine).ti,ab. | 10425460 |
| 28 | 26 not 27 | 685 |

| **Supplementary Table 2.** Embase (Ovid) database search | |  |
| --- | --- | --- |
| Database(s): Embase Classic+Embase 1947 to 2021 August 24 | |  |
| # | Searches | Results |
| 1 | exp electrocardiography/ | 201763 |
| 2 | (electrocardiogra* or "ECG*" or X-ECG or CPX or EGM or "Holter ECG" or "single averaged ECG*" or SAECG or "12-Lead ECG*" or "twelve lead ECG*").ti,ab,kw. | 233193 |
| 3 | ((cardi* or heart*) adj3 ("electrogra*" or "electro-gra*" or "exercis* test" or "stress test")).ti,ab,kw. | 6250 |
| 4 | ("T-wave alternans" or TWA or MWTA or "Index of Cardiac Electrophysiological Balance" or iCEB or "Heart rate variability" or HRV or "Heart rate turbulence" or HRT or "TpTe-interval" or TpTe or "Tpeak-Tend" or Tpe or "Tp-e" or "QT-dispersion*" or QTd or "QRS-duration*" or QRSd or "QT-interval" or "QT-time" or "Fragmented QRS" or fQRS or "global electrical heterogeneity" or "ventricular gradient*" or "spatial* ventricular gradient*" or "Early Repolarisation" or "QT Dynamicity").ti,ab,kw. | 81581 |
| 5 | or/1-4 | 404395 |
| 6 | exp artificial intelligence/ or exp machine learning/ | 287873 |
| 7 | ((machine or comput* or artificial* or deep or automat* or reinforce* or feature) adj3 (model* or intelligence or reasoning* or rational* or learn* or detect* or interpret* or algorithm* or simulat* or rank* or detect*)).ti,ab,kw. | 270585 |
| 8 | ("decision network" or "support vector machine*" or "vector machine*" or "deep architec*" or "convolution* neural network*" or "comput* vision" or "comput* aid* diagnos*" or "ensemble learning" or "ensemble method*" or "random forest*" or "random decision forest*" or "random survival* forest*" or ResNet or DenseNEt or "convolution network" or ANN or perceptron or radial basis or "k-Nearest Neighbor" or "k-nn" or "multilayer perceptron" or "recurrent neural network" or "Long short-term memory" or LSTM).ti,ab,kw. | 136131 |
| 9 | ((auto* or auto encod* or autoencod*) adj3 (quantif* or assess* or interpret* or identificat* or detect* or classif* or clust*)).ti,ab,kw. | 69636 |
| 10 | ((unsupervis* or supervis* or hierarch* or automat* or algorit* or ensemble or transfer*) adj3 (cluster* or classif* or learn*)).ti,ab,kw. | 81535 |
| 11 | ((neural or bayesian or "generative adversarial") adj3 (network* or learn*)).ti,ab,kw. | 86764 |
| 12 | or/6-11 | 634171 |
| 13 | 5 and 12 | 12415 |
| 14 | exp sudden cardiac death/ or exp heart arrest/ or exp heart arrhythmia/ or exp implantable cardioverter defibrillator/ or exp cardioversion/ | 659149 |
| 15 | ((ventricular* or cardiac or heart) adj3 (arrhythmia* or fibrillation or tachycardia or tachyarrhythmia* or flutter*)).ti,ab,kw. | 128457 |
| 16 | (sudden adj3 ((cardiac or heart) adj3 (arrest* or death*))).ti,ab,kw. | 33635 |
| 17 | (asystol* or "Cardiac Resynchronization Therapy Devices" or "CRT-D" or "implant* cardioverter-defibrillator*" or ICD* or "ICD therap*" or "ICD shock*" or "defibrillator shock*" or defibrillation* or "antitachycardia pac*" or "device* discharg*" or "electric* countershock*" or cardioversion* or "shock* rhythm*").ti,ab,kw. | 138119 |
| 18 | or/14-17 | 765744 |
| 19 | 13 and 18 | 3973 |
| 20 | exp prognosis/ or exp predictive value/ or exp receiver operating characteristic/ or exp risk assessment/ or exp incidence/ | 2166109 |
| 21 | ((predict* or risk* or prognos*) adj3 (recall or precision or outcome* or assess* or chart* or model* or test* or value* or variable* or score* or algorithm* or tool* or engine or equation* or calculat* or instrument* or method* or support* or rule*)).ti,ab,kw. | 1260281 |
| 22 | (prognos* or predict* or "area under the curve*" or "ROC Curve" or incidence or "youden index" or "youden statistic*" or "C statistic").ti,ab,kw. | 4276055 |
| 23 | ep.fs. | 1133775 |
| 24 | exp multicenter study/ or exp observational study/ or exp cohort analysis/ or exp longitudinal study/ or exp follow up/ or exp prospective study/ or exp retrospective study/ or exp validation study/ or (cohort* or longitudinal or prospective or retrospective or follow-up or follow up).ti,ab,kw. or (cohort adj3 (historic* or prospecti* or restrospec* or longitud* or follow-up or follow up or stud*)).ti,ab,kw. or ("validat* stud*" or observat* stud* or "replicat* stud*").ti,ab. or (cohort stud* or validation stud* or longitudinal stud* or prospec stud* or resrospective stud* or validation stud*).pt. | 5233159 |
| 25 | or/20-24 | 9286400 |
| 26 | 19 and 25 | 1890 |
| 27 | ((exp animals/ or exp veterinary medicine/ or animal*.jw.) not humans/) or (experimental model or animal* or monkey* or sheep or ovine or lamb or lambs or goat* or pig or pigs or swine or porcine or pup or pups or dog or dogs or canine or bitch* or beagle or feline or rodent* or rabbit* or rat or rats or mouse or murine).ti,ab. | 14025068 |
| 28 | 26 not 27 | 1208 |

| **Supplementary Table 3.** Scopus 1947 to 2021 August 24 | |
| --- | --- |
| Searches | Results |
| TITLE-ABS-KEY-AUTH(("T-wave alternans" or TWA or MWTA or "Index of Cardiac Electrophysiological Balance" or iCEB or "Heart rate variability" or HRV or "Heart rate turbulence" or HRT or "TpTe-interval" or TpTe or "Tpeak-Tend" or Tpe or "Tp-e" or "QT-dispersion*" or QTd or "QRS-duration*" or QRSd or "QT-interval" or "QT-time" or "Fragmented QRS" or fQRS or "global electrical heterogeneity" or "ventricular gradient*" or "spatial* ventricular gradient*" or "Early Repolarisation" or "QT Dynamicity" or electrocardiogra* or "ECG*" or X-ECG or CPX or EGM or "Holter ECG" or "single averaged ECG*" or SAECG or "12-Lead ECG*" or "twelve lead ECG*") or ((cardi* or heart*) W/3 ("electrogra*" or "electro-gra*" or "exercis* test" or "stress test"))) AND TITLE-ABS-KEY-AUTH(((machine or comput* or artificial* or deep or automat* or reinforce* or feature) W/3 (model* or intelligence or reasoning* or rational* or learn* or detect* or interpret* or algorithm* or simulat* or rank* or detect*)) or ((unsupervis* or supervis* or hierarch* or automat* or algorit* or ensemble or transfer*) W/3 (cluster* or classif* or learn*)) or ((neural or bayesian or "generative adversarial") W/3 (network* or learn*)) or ("decision network" or "support vector machine*" or "vector machine*" or "deep architec*" or "convolution* neural network*" or "comput* vision" or "comput* aid* diagnos*" or "ensemble learning" or "ensemble method*" or "random forest*" or "random decision forest*" or "random survival* forest*" or ResNet or DenseNEt or "convolution network" or ANN or perceptron or radial basis or "k-Nearest Neighbor" or "k-nn" or "multilayer perceptron" or "recurrent neural network" or "Long short-term memory" or LSTM)) AND TITLE-ABS-KEY-AUTH(((ventricular* or cardiac or heart) W/3 (arrhythmia* or fibrillation or tachycardia or tachyarrhythmia* or flutter*)) or (sudden W/3 ((cardiac or heart) W/3 (arrest* or death*))) or (asystol* or "Cardiac Resynchronization Therapy Devices" or "CRT-D" or "implant* cardioverter-defibrillator*" or ICD* or "ICD therap*" or "ICD shock*" or "defibrillator shock*" or defibrillation* or "antitachycardia pac*" or "device* discharg*" or "electric* countershock*" or cardioversion* or "shock* rhythm*")) AND TITLE-ABS-KEY-AUTH(((predict* or risk* or prognos*) W/3 (recall or precision or outcome* or assess* or chart* or model* or test* or value* or variable* or score* or algorithm* or tool* or engine or equation* or calculat* or instrument* or method* or support* or rule*)) or (prognos* or predict* or "area under the curve*" or "ROC Curve" or incidence or "youden index" or "youden statistic*" or "C statistic")) AND NOT TITLE-ABS-KEY-AUTH("veterinary medicine" or animal* or monkey* or sheep or ovine or lamb* or goat* or pig* or swine* or porcine or pup* or dog* or canine* or bitch* or beagle* or feline* or rodent* or rabbit* or rat* or mice or mouse or murine) | 587 |

| **Supplementary Table 4.** Web of Science 1947 to 2021 August 24 | | |
| --- | --- | --- |
| Searches | | Results |
| Query #1 | (TS=("T-wave alternans" or TWA or MWTA or "Index of Cardiac Electrophysiological Balance" or iCEB or "Heart rate variability" or HRV or "Heart rate turbulence" or HRT or "TpTe-interval" or TpTe or "Tpeak-Tend" or Tpe or "Tp-e" or "QT-dispersion*" or QTd or "QRS-duration*" or QRSd or "QT-interval" or "QT-time" or "Fragmented QRS" or fQRS or "global electrical heterogeneity" or "ventricular gradient*" or "spatial* ventricular gradient*" or "Early Repolarisation" or "QT Dynamicity" or electrocardiogra* or "ECG*" or X-ECG or CPX or EGM or "Holter ECG" or "single averaged ECG*" or SAECG or "12-Lead ECG*" or "twelve lead ECG*")) OR TS=(((cardi* or heart*) NEAR/3 ("electrogra*" or "electro-gra*" or "exercis* test" or "stress test"))) |  |
| Query #2 | TS=(((machine or comput* or artificial* or deep or automat* or reinforce* or feature) NEAR/3 (model* or intelligence or reasoning* or rational* or learn* or detect* or interpret* or algorithm* or simulat* or rank* or detect*)) or ((unsupervis* or supervis* or hierarch* or automat* or algorit* or ensemble or transfer*) NEAR/3 (cluster* or classif* or learn*)) or ((neural or bayesian or "generative adversarial") NEAR/3 (network* or learn*)) or ("decision network" or "support vector machine*" or "vector machine*" or "deep architec*" or "convolution* neural network*" or "comput* vision" or "comput* aid* diagnos*" or "ensemble learning" or "ensemble method*" or "random forest*" or "random decision forest*" or "random survival* forest*" or ResNet or DenseNEt or "convolution network" or ANN or perceptron or radial basis or "k-Nearest Neighbor" or "k-nn" or "multilayer perceptron" or "recurrent neural network" or "Long short-term memory" or LSTM)) |  |
| Query #3 | TS=(((ventricular* or cardiac or heart) NEAR/3 (arrhythmia* or fibrillation or tachycardia or tachyarrhythmia* or flutter*)) or (sudden NEAR/3 ((cardiac or heart) NEAR/3 (arrest* or death*))) or (asystol* or "Cardiac Resynchronization Therapy Devices" or "CRT-D" or "implant* cardioverter-defibrillator*" or ICD* or "ICD therap*" or "ICD shock*" or "defibrillator shock*" or defibrillation* or "antitachycardia pac*" or "device* discharg*" or "electric* countershock*" or cardioversion* or "shock* rhythm*")) |  |
| Query #4 | TS=(((predict* or risk* or prognos*) NEAR/3 (recall or precision or outcome* or assess* or chart* or model* or test* or value* or variable* or score* or algorithm* or tool* or engine or equation* or calculat* or instrument* or method* or support* or rule*)) or (prognos* or predict* or "area under the curve*" or "ROC Curve" or incidence or "youden index" or "youden statistic*" or "C statistic")) |  |
|  | (((#1) AND #2) AND #3) AND #4 | 457 |

| **Supplementary Table 5.** Cochrane 1947 to 2021 August 24 | |
| --- | --- |
| Searches | Results |
| machine learning |  |
| AND |  |
| sudden cardiac arrest or "sudden cardiac death" or "ventricular arrhythmia" |  |
|  | 6 trials |

| **Supplementary Table 6.** Details regarding the electrophysiological signals used by models (n=10) developed on clinically-defined datasets | | | | | | |
| --- | --- | --- | --- | --- | --- | --- |
| **#** | **Author** | **Recording** | **Recording duration** | **ECG leads** | **Sample** | **Prediction window** |
| 1 | Au-Yeung et al. (1) | EGM | 25-30 minutes (2048 R-R prior to shock) | N/A | 128 Hz | 5 minutes prior to event |
| 2 | Do et al. (2) | ECG | Continuous | 7-lead | 240 Hz | 1-hour long windows prior to event |
| 3 | Lee et al. (3) | ECG | Continuous | 1-lead | Unknown | 1 hour prior to event |
| 4 | Kwon et al. (4) | ECG | 10 sec | 12-lead | 500 Hz | 24 hours prior to event |
| 5 | Gleeson et al. (5) | ECG | 10 sec | 12-lead | unknown | 21 months |
| 6 | Martinez-Alanis et al. (6) | EGM | 15 minutes | N/A | unknown | 1 minute |
| 7 | Ong et al. (7) | ECG | 5-30 min | 12-lead (lead II as input) | 125 Hz | 72 hours |
| 8 | Ramirez et al. (8) | ECG | 24 hour | 2 or 3-lead | 200 Hz | Median 44 months |
| 9 | Rodriguez et al. (9) | ECG | 30 minutes | Unknown | 1.600Hz | Median 28 months (IQR 17–38 months) |
| 10 | Rogers et al. (10) | Signals obtained during electrophysiological studies | N/A | N/A |  | Median 41.6 months (IQR 29.7–77.7) |
| *Abbreviations*: ECG=electrocardiography, EGM=intracardiac electrogram, IQR=interquartile range, PVC=premature ventricular complex, SCD=sudden cardiac death | | | | | | |

| **Supplementary Table 7.** Features extracted from electrophysiological signals and used as input for the ML and DL models | | |
| --- | --- | --- |
| **Time-domain** | | |
|  | Direct measurement of RR interval(1, 3, 6, 7, 11-27) | - Mean NN, Mean of all RR intervals - SDNN: standard deviation of all RR intervals - Ratio Mean NN/ SDNN - MeanHR: mean value of heart rate - sdHR: standard deviation of heart rate |
|  | Difference between RR intervals(1, 3, 6, 7, 11-27) | - RMSSD: The square root of the mean of the sum of the squares of differences between adjacent NN Intervals. - SDSD: The standard deviation of differences between adjacent NN intervals. - PNN50: proportion of interval differences of NN intervals greater than 50 ms |
|  | Geometric-domain(7, 22) | - HRV triangular index (Total number of all RR intervals divided by the height of the histogram of intervals) - TINN (Baseline width of a triangle fit into the RR interval histogram using a least squares technique |
| **Frequency-domain** | | |
|  | Total energy of signal per frequency band (relative, absolute) (1, 3, 6, 7, 11-14, 16-19, 22-25, 28) | - Power of the very low frequency band (0.001-0.04 Hz) - Power of the Low frequency band (0.04-0.15 Hz) - Power of the high frequency band (0.15-0.4 Hz), - Power of the LF/HF (ratio) |
| **Time-Frequency domain** | | |
|  | Non-parametric linear TF representation based on linear filtering | Fast Fourier Transform(28) |
|  |  | Discrete Wavelet Transform (22, 29-31) |
|  |  | Wavelet packet transform (32) |
|  |  | Continuous wavelet transform (11, 33, 34) |
|  |  | Empirical mode decomposition (24, 35) |
|  | Nonparametric quadratic TF representation | Smoothed Pseudo Wigner Ville distribution (12, 17, 18, 23) |
| **Non-linear domain** | | |
|  | Fractal | Detrended fluctuation analysis (1, 17, 19, 22, 29) |
|  |  | Fractal dimensions: (29, 35, 36)   - Katz FD - Higuchi FD - Box dimension |
|  |  | Hurst exponent (29, 37) |
|  |  | Correlation dimensions(29) |
|  | Information/Entropy(1, 22, 24, 29, 31, 35, 37, 38) | - Sample entropy - Approximate entropy - Largest Lyapunov Exponent - Renyi entropy - Fuzzy entropy - Hjorth’s parameters (activity, mobility, complexity) - Tsallis entropy - Increment entropy |
|  | Representation methods | Poincaré plot: (3, 9, 12, 13, 16, 17, 19, 22, 23, 25, 39)   - SD1: Standard deviation of the short-term RR interval variability - SD2: standard deviation of the long-term RR interval variability - SD1/SD2: ratio |
|  |  | Recurrence plot: (27, 30, 38, 39)   - RQA parameters |
|  | Kolmogorov complexity(30) | Quantify the complexity and irregularity of HRV signals |
|  | Higher order spectral (HOS) features(20, 25) | The magnitude average, power average, normalized and square normalized entropies, logarithmic bispectrum features, and weight center of bispectrum |
|  | Homogeneity index(32) |  |
|  | Phase space portraits(40) | Mean, SD, variation, skewness, kurtosis |
| **Morphology and intervals** | | |
|  | QRS complex features and ventricular repolarisation feature(5, 8, 13-15, 33, 41-45) (2, 8, 33) | - Segments (QRS, QT, JT_peak_, JT_end_, T_p_T_e_/QT): mean value, standard deviation, average mean root square - Amplitude (Q, R, S): absolute value, min-max, standard deviation - Turbulence slope, T-wave alternans (magnitude, interval), dispersion in repolarisation restitution |
| **Miscellaneous** | | |
|  | Mathematical features | Frequency domain and mathematical indices of action potential shape (10) |
|  | Heart print indices | PVCs/hour, mean CI, SDCI, sNIB, NIB mode(6) |
| *Abbreviations*: CI=confidence interval, HRV=heart rate variability, NIB= number of sinus intervening beats, meanCI=mean coupling interval, PVC=premature ventricular complex, sNIB=NIB score, | | |

***Supplementary Figure 1.*** Risk of bias assessment of studies (n=10) developed using a clinically-defined dataset according to *PROBAST: A Tool to Assess the Risk of Bias and Applicability of Prediction Model Studies*****

***Supplementary Figure 2.*** Risk of bias assessment of studies (n=36) developed using ad-hoc datasets according to *PROBAST: A Tool to Assess the Risk of Bias and Applicability of Prediction Model Studies*

***Supplementary Figure 3.*** Forest model of the specificity (Panel 3a) and sensitivity (Panel 3b), and the 95% confidence interval of models developed to predict on a short horizon (within 72 hours)


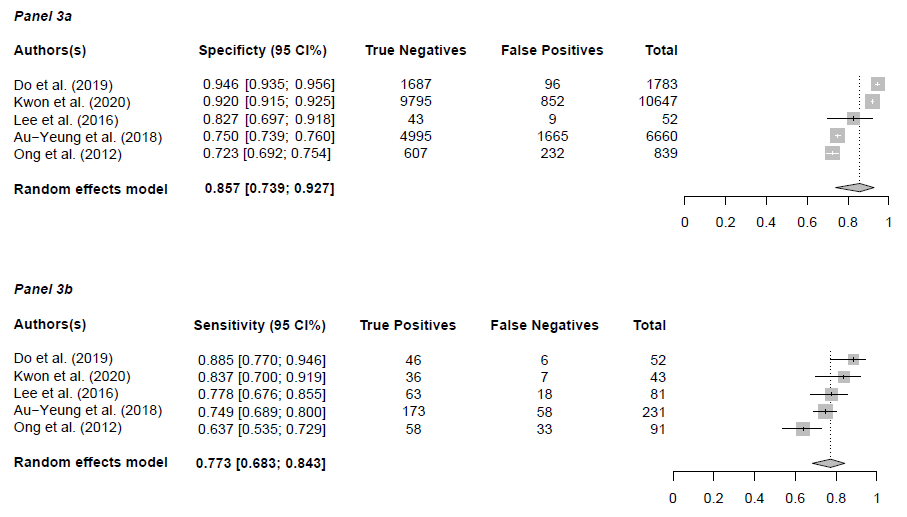


***Supplementary Figure 4.*** Forest model of the specificity (Panel 4a) and sensitivity (Panel 4b), and the 95% confidence interval of models developed to predict on a long horizon


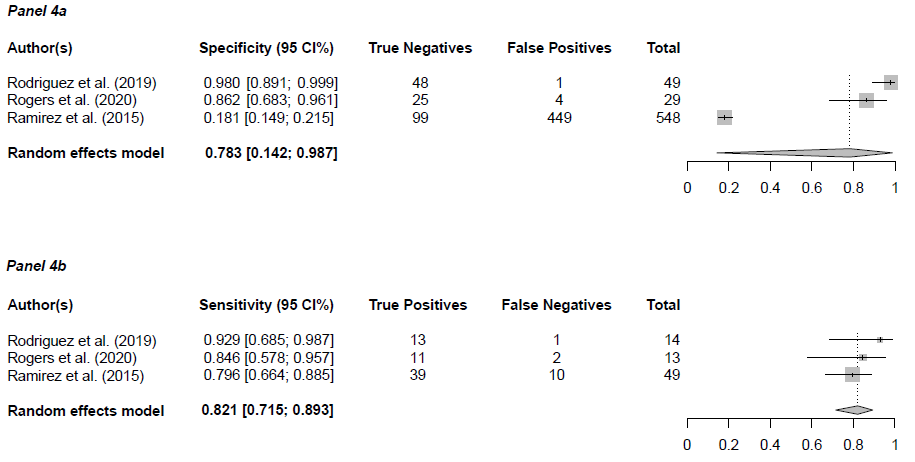


***Supplementary Figure 5.*** Forest model of the diagnostic odds ratio (DOR) and 95% confidence interval for low and high risk of bias


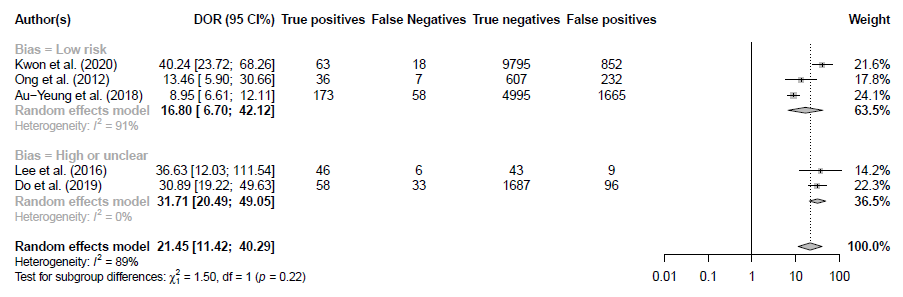


***Supplementary Figure 6.*** Forest model of the diagnostic odds ratio (DOR) and 95% confidence interval for sample sizes <500 and ≥500


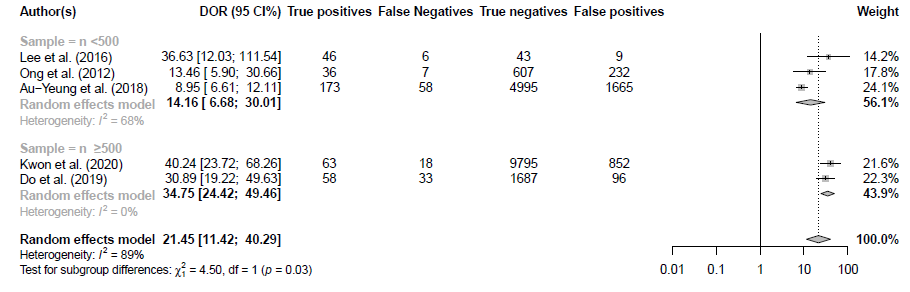


***Supplementary Figure 7.*** Leave-one-out sensitivity analysis of models developed to predict on a short horizon (Panel 7a) and long horizon (Panel 7b)


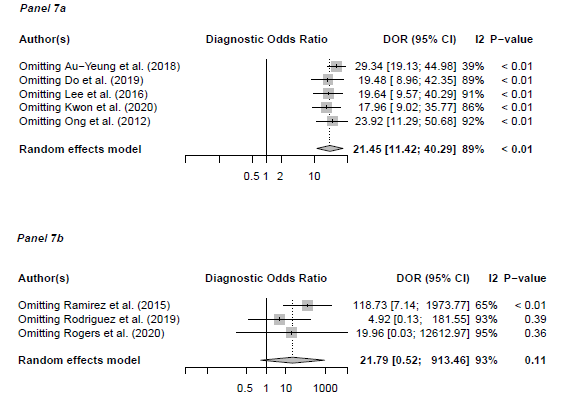


***Supplementary Figure 8.*** Funnel plot for models developed to predict on a short horizon (Panel 8a), and combined short and long horizon (Panel 8b). Grey markers represent original studies, white markers represent filled studies.
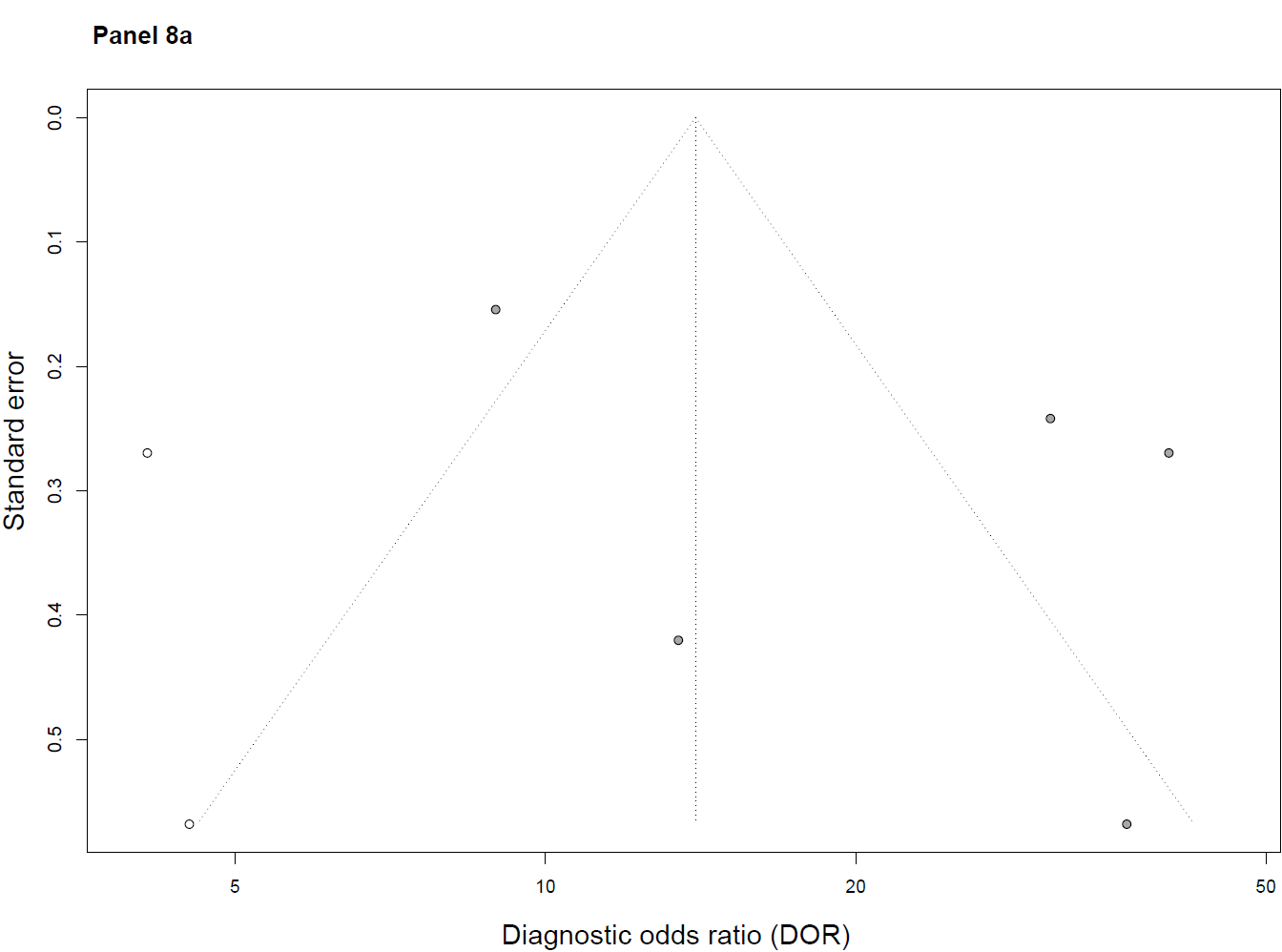


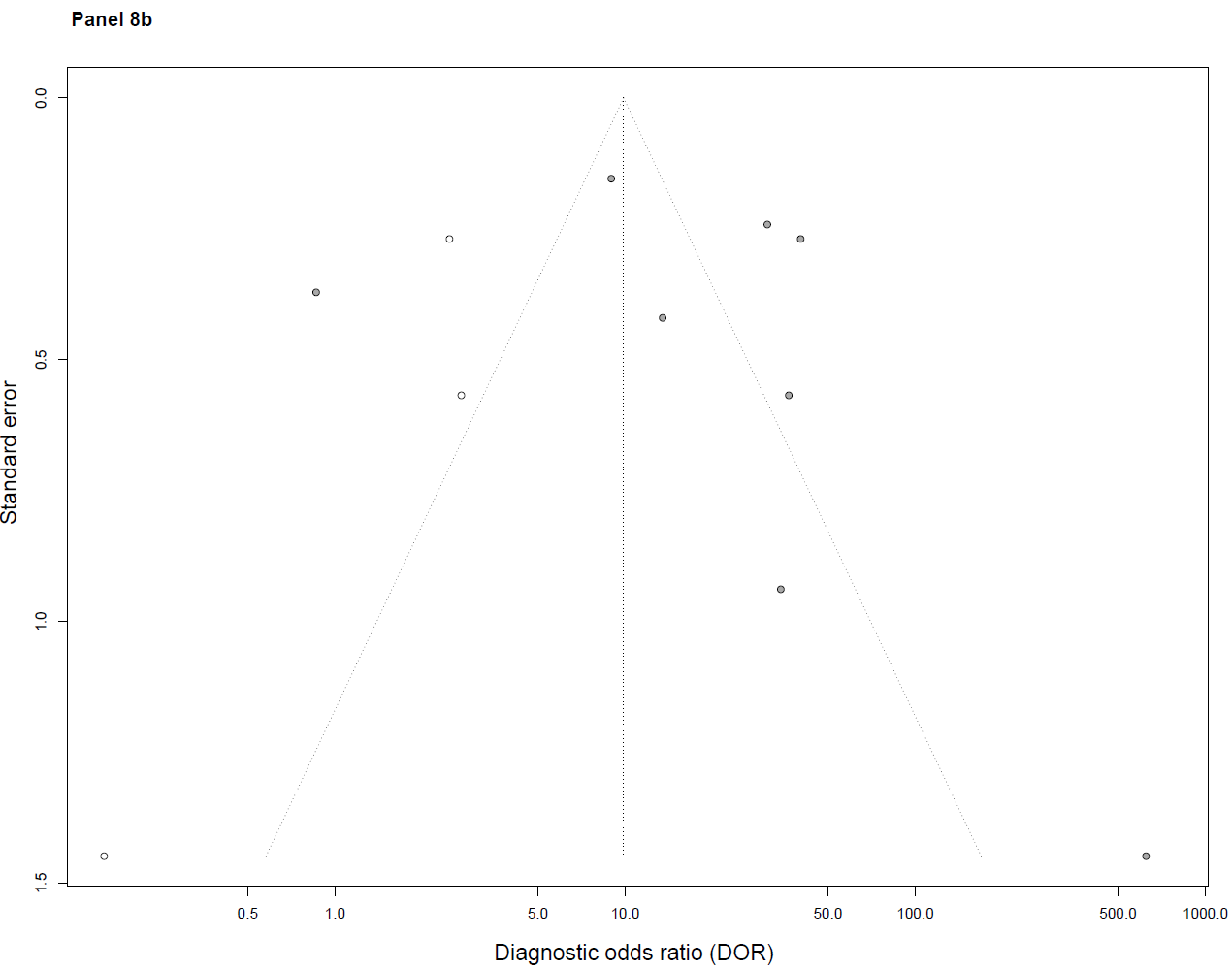


***Supplementary Figure 9.*** Forest model of the specificity (Panel 9a) and sensitivity (Panel 9b), and the 95% confidence interval of models developed using ad-hoc datasets


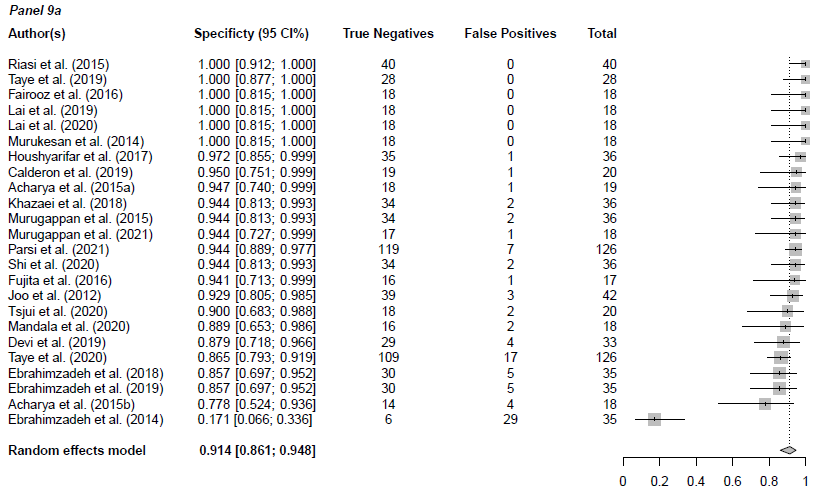


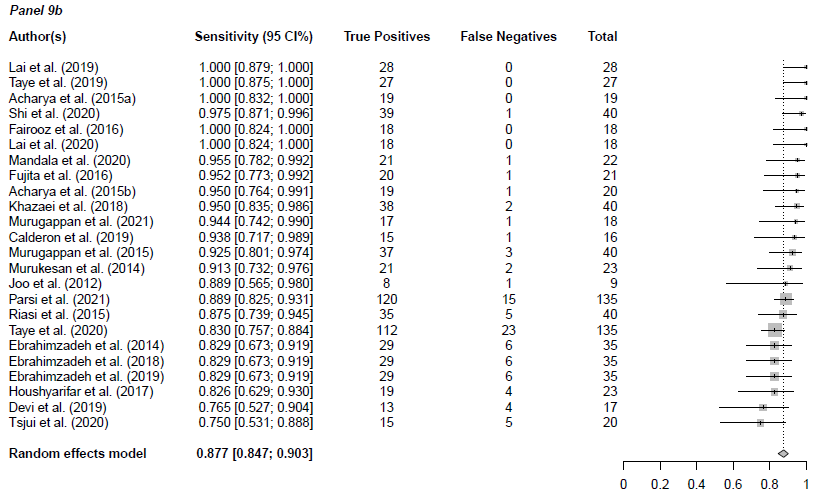


***Supplementary Figure 10.*** Forest plot of diagnostic odds ratio (DOR) and 95% confidence interval for models developed using ad-hoc datasets


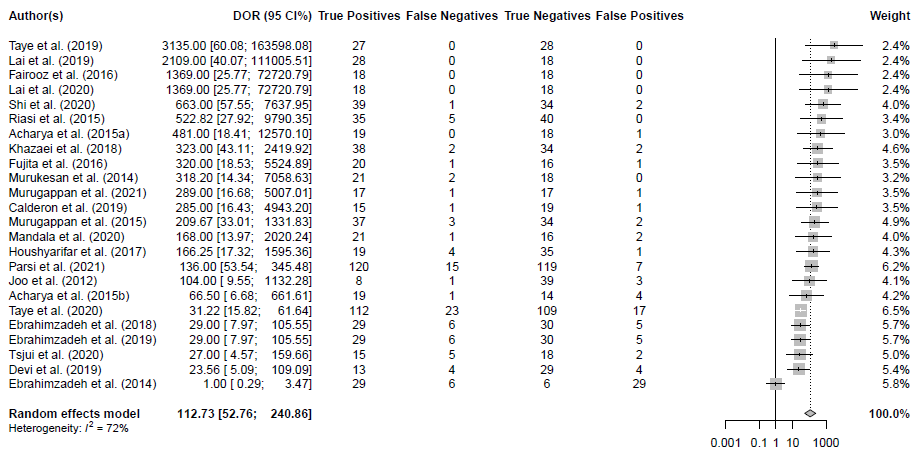


***Supplementary Figure 11.*** Forest plot of diagnostic odds ratio (DOR) and 95% confidence interval for models developed using ad-hoc datasets per ad-hoc dataset or combination of datasets
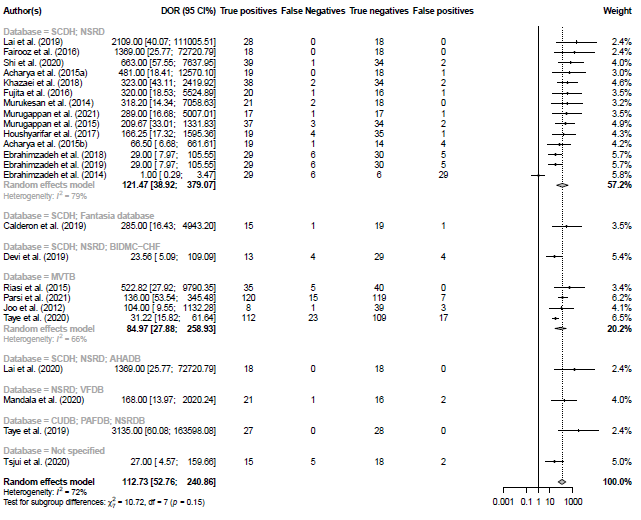


***Supplementary Figure 12.*** Forest plot of diagnostic odds ratio (DOR) and 95% confidence interval for models developed using ad-hoc datasets per year of publication


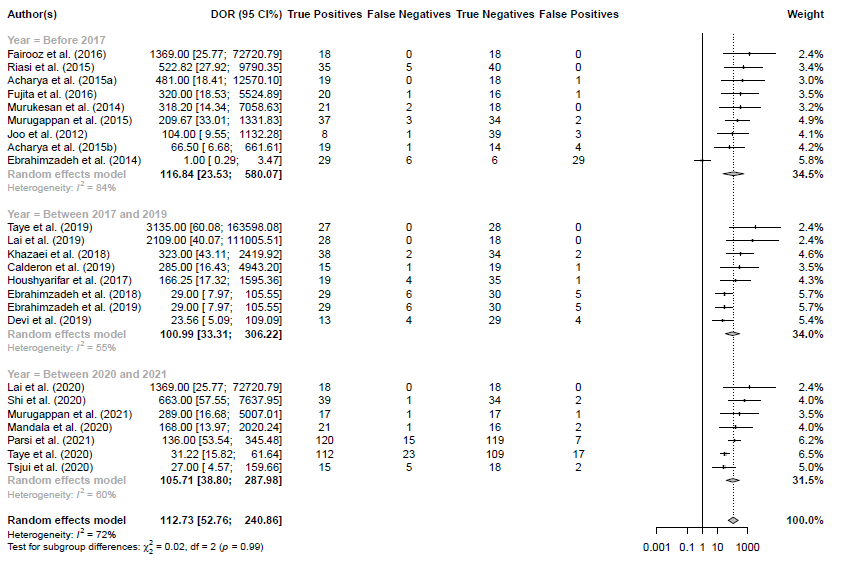


***Supplementary Figure 13.*** Forest plot of diagnostic odds ratio (DOR) and 95% confidence interval for models developed using ad-hoc datasets per risk of bias


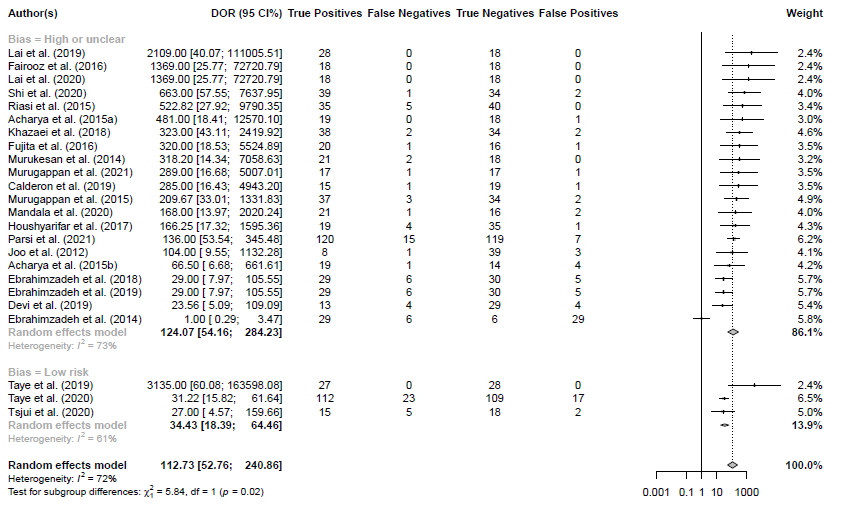
***Supplementary Figure 14.*** Forest plot of diagnostic odds ratio (DOR) and 95% confidence interval for models developed using ad-hoc datasets per region of origin


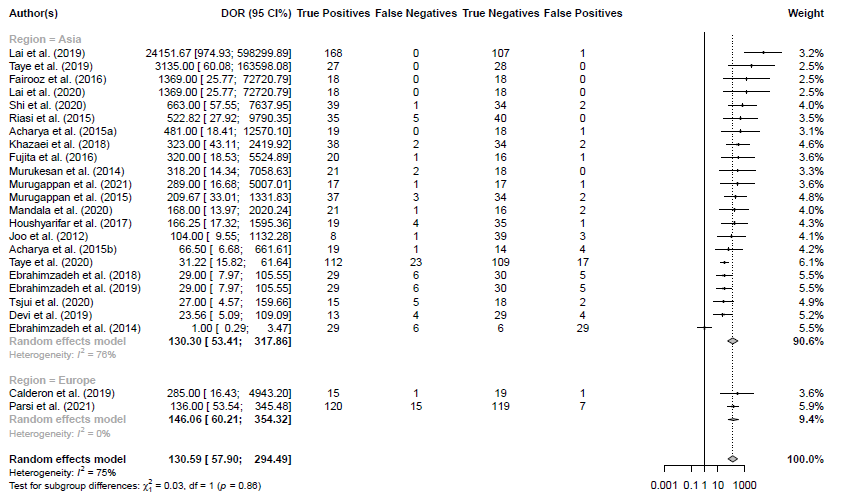


***Supplementary Figure 15.*** Leave-one-out sensitivity analysis of models developed using ad-hoc datasets


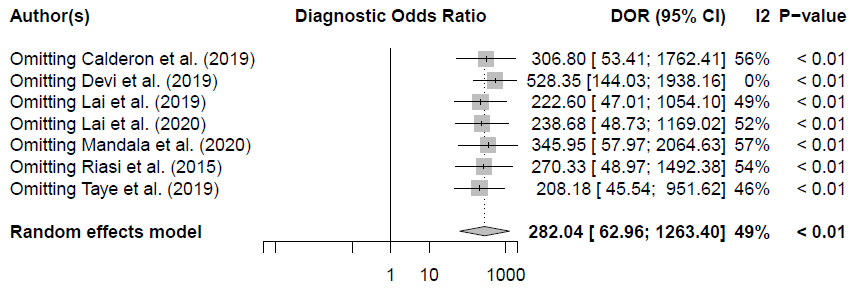


***Supplementary Figure 16.*** Funnel plot for models developed using ad-hoc dataset. Grey markers represent original studies, white markers represent filled studies.


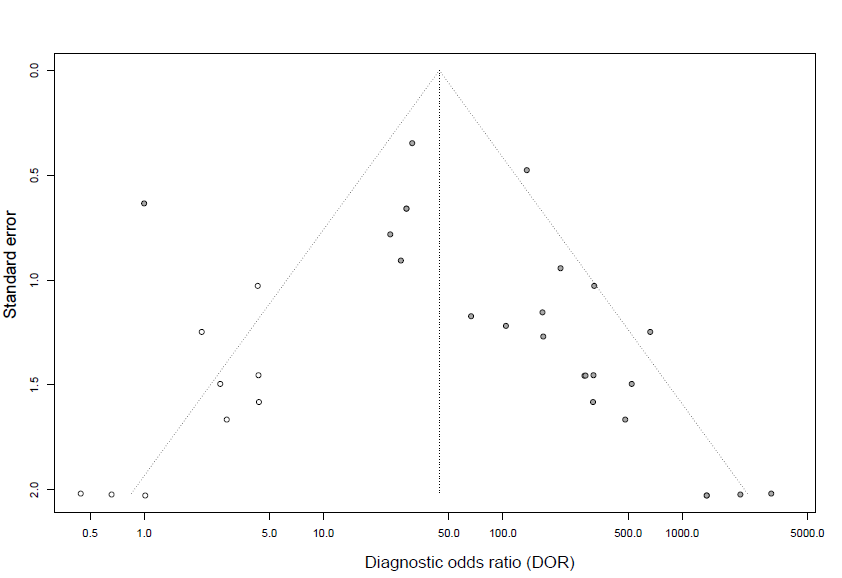


***Supplementary Figure 17.*** Bubble plot of the diagnostic odds ratio (DOR) for models developed using ad-hoc databases per type of algorithm
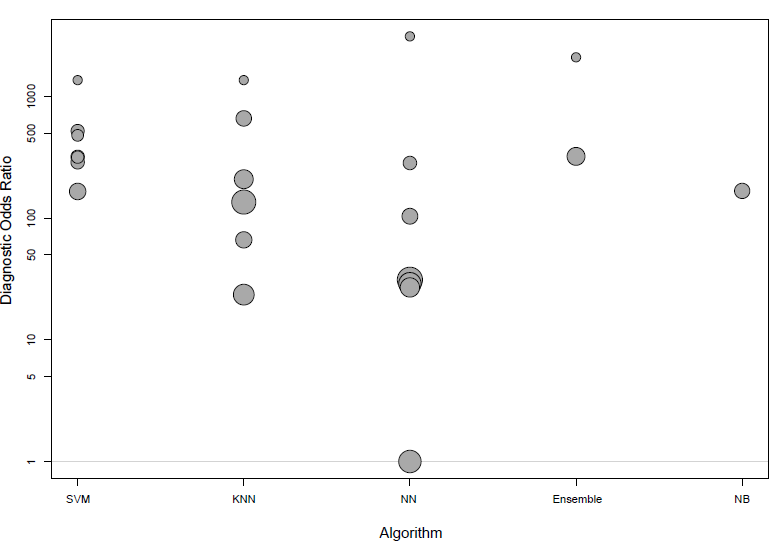


**References**

1. Au-Yeung WM, Reinhall PG, Bardy GH, Brunton SL. Development and validation of warning system of ventricular tachyarrhythmia in patients with heart failure with heart rate variability data. PLoS One. 2018;13(11):e0207215.

2. Do DH, Kuo A, Lee ES, Mortara D, Elashoff D, Hu X, et al. Usefulness of Trends in Continuous Electrocardiographic Telemetry Monitoring to Predict In-Hospital Cardiac Arrest. Am J Cardiol. 2019;124(7):1149-58.

3. Lee H, Shin SY, Seo M, Nam GB, Joo S. Prediction of Ventricular Tachycardia One Hour before Occurrence Using Artificial Neural Networks. Sci Rep. 2016;6:32390.

4. Kwon JM, Kim KH, Jeon KH, Lee SY, Park J, Oh BH. Artificial intelligence algorithm for predicting cardiac arrest using electrocardiography. Scand J Trauma Resusc Emerg Med. 2020;28(1):98.

5. Gleeson S, Liao YW, Dugo C, Cave A, Zhou L, Ayar Z, et al. ECG-derived spatial QRS-T angle is associated with ICD implantation, mortality and heart failure admissions in patients with LV systolic dysfunction. PLoS One. 2017;12(3):e0171069.

6. Martinez-Alanis M, Bojorges-Valdez E, Wessel N, Lerma C. Prediction of Sudden Cardiac Death Risk with a Support Vector Machine Based on Heart Rate Variability and Heartprint Indices. Sensors (Basel). 2020;20(19).

7. Ong MEH, Lee Ng CH, Goh K, Liu N, Koh Z, Shahidah N, et al. Prediction of cardiac arrest in critically ill patients presenting to the emergency department using a machine learning score incorporating heart rate variability compared with the modified early warning score. Critical Care. 2012;16(3).

8. Ramirez J, Monasterio V, Minchole A, Llamedo M, Lenis G, Cygankiewicz I, et al. Automatic SVM classification of sudden cardiac death and pump failure death from autonomic and repolarization ECG markers. J Electrocardiol. 2015;48(4):551-7.

9. Rodriguez J, Schulz S, Giraldo BF, Voss A. Risk Stratification in Idiopathic Dilated Cardiomyopathy Patients Using Cardiovascular Coupling Analysis. Front Physiol. 2019;10:841.

10. Rogers AJ, Selvalingam A, Alhusseini MI, Krummen DE, Corrado C, Abuzaid F, et al. Machine Learned Cellular Phenotypes in Cardiomyopathy Predict Sudden Death. Circ Res. 2021;128(2):172-84.

11. Devi R, Tyagi HK, Kumar D. A novel multi-class approach for early-stage prediction of sudden cardiac death. Biocybernetics and Biomedical Engineering. 2019;39(3):586-98.

12. Mirhoseini Seyyed Rohollah MRJ, Mohammad Pooyan. Improve Accuracy of Early Detection Sudden Cardiac Deaths (SCD) Using Decision Forest and SVM. International Conference on Robotics and Artificial Intelligence 2016. 2016.

13. Taye GT, Shim EB, Hwang HJ, Lim KM. Machine Learning Approach to Predict Ventricular Fibrillation Based on QRS Complex Shape. Front Physiol. 2019;10:1193.

14. Alfarhan KA, Mashor MY, Zakaria A, Omar MI. Automated Electrocardiogram Signals Based Risk Marker for Early Sudden Cardiac Death Prediction. Journal of Medical Imaging and Health Informatics. 2018;8(9):1769-75.

15. Mandala S, Cai Di T, Sunar MS, Adiwijaya. ECG-based prediction algorithm for imminent malignant ventricular arrhythmias using decision tree. PLoS One. 2020;15(5):e0231635.

16. Joo S, Choi K-J, Huh S-J. Prediction of spontaneous ventricular tachyarrhythmia by an artificial neural network using parameters gleaned from short-term heart rate variability. Expert Systems with Applications. 2012;39(3):3862-6.

17. Ebrahimzadeh E, Pooyan M, Bijar A. A novel approach to predict sudden cardiac death (SCD) using nonlinear and time-frequency analyses from HRV signals. PLoS One. 2014;9(2):e81896.

18. Ebrahimzadeh E, Pooyan M. Early detection of sudden cardiac death by using classical linear techniques and time-frequency methods on electrocardiogram signals. Journal of Biomedical Science and Engineering. 2011;04(11):699-706.

19. Ebrahimzadeh E, Manuchehri MS, Amoozegar S, Araabi BN, Soltanian-Zadeh H. A time local subset feature selection for prediction of sudden cardiac death from ECG signal. Med Biol Eng Comput. 2018;56(7):1253-70.

20. Houshyarifar V, Chehel Amirani M. An approach to predict Sudden Cardiac Death (SCD) using time domain and bispectrum features from HRV signal. Biomed Mater Eng. 2016;27(2-3):275-85.

21. Tsuji T, Nobukawa T, Mito A, Hirano H, Soh Z, Inokuchi R, et al. Recurrent probabilistic neural network-based short-term prediction for acute hypotension and ventricular fibrillation. Sci Rep. 2020;10(1):11970.

22. Murukesan L, Murugappan M, Iqbal M, Saravanan K. Machine Learning Approach for Sudden Cardiac Arrest Prediction Based on Optimal Heart Rate Variability Features. Journal of Medical Imaging and Health Informatics. 2014;4(4):521-32.

23. Ebrahimzadeh E, Foroutan A, Shams M, Baradaran R, Rajabion L, Joulani M, et al. An optimal strategy for prediction of sudden cardiac death through a pioneering feature-selection approach from HRV signal. Comput Methods Programs Biomed. 2019;169:19-36.

24. Shi M, He H, Geng W, Wu R, Zhan C, Jin Y, et al. Early Detection of Sudden Cardiac Death by Using Ensemble Empirical Mode Decomposition-Based Entropy and Classical Linear Features From Heart Rate Variability Signals. Front Physiol. 2020;11:118.

25. Parsi A, Byrne D, Glavin M, Jones E. Heart rate variability feature selection method for automated prediction of sudden cardiac death. Biomedical Signal Processing and Control. 2021;65.

26. Murugappan M, Murukesan L, Omar I, Khatun S, Murugappan S. Time Domain Features Based Sudden Cardiac Arrest Prediction Using Machine Learning Algorithms. Journal of Medical Imaging and Health Informatics. 2015;5(6):1267-71.

27. Jeong DU, Taye GT, Hwang H-J, Lim KM, Martínez JP. Optimal Length of Heart Rate Variability Data and Forecasting Time for Ventricular Fibrillation Prediction Using Machine Learning. Computational and Mathematical Methods in Medicine. 2021;2021:1-5.

28. Shen T.S. SH, Lin C. and Ou Y. Detection and Prediction of Sudden Cardiac Death (SCD) For Personal Healthcare. Proceedings of the 29th Annual International Conference of the IEEE EMBS. 2006.

29. Acharya UR, Fujita H, Sudarshan VK, Sree VS, Eugene LWJ, Ghista DN, et al. An integrated index for detection of Sudden Cardiac Death using Discrete Wavelet Transform and nonlinear features. Knowledge-Based Systems. 2015;83:149-58.

30. Acharya UR, Fujita H, Sudarshan VK, Ghista DN, Lim WJE, Koh JEW. Automated Prediction of Sudden Cardiac Death Risk Using Kolmogorov Complexity and Recurrence Quantification Analysis Features Extracted from HRV Signals. 2015 IEEE International Conference on Systems, Man, and Cybernetics2015. p. 1110-5.

31. Fujita H, Acharya UR, Sudarshan VK, Ghista DN, Sree SV, Eugene LWJ, et al. Sudden cardiac death (SCD) prediction based on nonlinear heart rate variability features and SCD index. Applied Soft Computing. 2016;43:510-9.

32. Amezquita-Sanchez JP, Valtierra-Rodriguez M, Adeli H, Perez-Ramirez CA. A Novel Wavelet Transform-Homogeneity Model for Sudden Cardiac Death Prediction Using ECG Signals. J Med Syst. 2018;42(10):176.

33. Fairooz T, Khammari H. SVM classification of CWT signal features for predicting sudden cardiac death. Biomedical Physics & Engineering Express. 2016;2(2).

34. Tseng L-M, Tseng VS. Predicting Ventricular Fibrillation Through Deep Learning. IEEE Access. 2020;8:221886-96.

35. Vargas-Lopez O, Amezquita-Sanchez JP, De-Santiago-Perez JJ, Rivera-Guillen JR, Valtierra-Rodriguez M, Toledano-Ayala M, et al. A New Methodology Based on EMD and Nonlinear Measurements for Sudden Cardiac Death Detection. Sensors (Basel). 2019;20(1).

36. Lopez-Caracheo C, Perez-Ramirez. Fractal Dimension-based Methodology for Sudden Cardiac Death Prediction. 2018.

37. Murugappan M, Murugesan L, Jerritta S, Adeli H. Sudden Cardiac Arrest (SCA) Prediction Using ECG Morphological Features. Arabian Journal for Science and Engineering. 2020;46(2):947-61.

38. Khazaei M, Raeisi K, Goshvarpour A, Ahmadzadeh M. Early detection of sudden cardiac death using nonlinear analysis of heart rate variability. Biocybernetics and Biomedical Engineering. 2018;38(4):931-40.

39. Houshyarifar V, Amirani MC. Early detection of sudden cardiac death using Poincaré plots and recurrence plot-based features from HRV signals. Turkish Journal of Electrical Engineering & Computer Sciences. 2017;25:1541-53.

40. Cappiello G, Das S, Mazomenos EB, Maharatna K, Koulaouzidis G, Morgan J, et al. A statistical index for early diagnosis of ventricular arrhythmia from the trend analysis of ECG phase-portraits. Physiol Meas. 2015;36(1):107-31.

41. Atiye Riasi MM. Prediction Of Ventricular Tachycardia Using Morphological Features Of ECG Signal.

42. Bayasi N, Tekeste T, Saleh H, Khandoker AH, Mohammad B, Ismail M. A novel algorithm for the prediction and detection of ventricular arrhythmia. Analog Integrated Circuits and Signal Processing. 2019;99(2):413-26.

43. Calderon A, Perez A, Valente J. ECG Feature Extraction and Ventricular Fibrillation (VF) Prediction using Data Mining Techniques. 2019 IEEE 32nd International Symposium on Computer-Based Medical Systems (CBMS)2019. p. 14-9.

44. Lai D, Zhang Y, Zhang X, Su Y, Bin Heyat MB. An Automated Strategy for Early Risk Identification of Sudden Cardiac Death by Using Machine Learning Approach on Measurable Arrhythmic Risk Markers. IEEE Access. 2019;7:94701-16.

45. Lai DaZ, Y. and Zhang, X. Single Lead ECG-based Ventricular Repolarization Classification for Early Identification of Unexpected Ventricular Fibrillation. Annual International Conference Of The IEEE Engineering In Medicine And Biology Society. 2020.
